# Spatial and single-cell transcriptomics uncover brassinosteroid-mediated coordination of sepal elongation in Arabidopsis

**DOI:** 10.1186/s13059-026-04156-1

**Published:** 2026-06-17

**Authors:** Byron Rusnak, Lilijana Sarabia Oliver, Kyle Procopio, Adrienne H. K. Roeder

**Affiliations:** 1 Weill Institute for Cell and Molecular Biology, Cornell University, Ithaca, NY 14853 USA; 2 Section of Plant Biology, School of Integrative Plant Science, Cornell University, Ithaca, NY 14853 USA

**Keywords:** Inter-organ growth coordination, *Arabidopsis thaliana*, Sepal, Brassinosteroids, Single cell RNA-seq, Spatial transcriptomics

## Abstract

**Background:**

*Arabidopsis* sepals must grow in a coordinated and robust manner to achieve consistent size and shape, ensuring proper closure and protecting the developing flower bud. To understand how this robust coordination occurs, we analyze the loss-of-robustness mutant *development related myb-like1* (*drmy1*), which exhibits variable sepal initiation and growth, causing failure of bud closure. Specifically, *drmy1* displays disproportionate sepal growth, with overgrown outer sepals and undergrown inner sepals, resulting in marked size variability within individual flower buds.

**Results:**

Using single cell and spatial RNA-seq, we identify changes in expression of key genes related to brassinosteroid (BR) signaling in *drmy1*, particularly in cell types important to early flower bud development such as epidermal cells, boundary cells, and meristematic cells. Confocal imaging of a BRI1-EMS-SUPPRESSOR1 (BES1) ratiometric reporter confirms that BR signaling is upregulated and more variable in young *drmy1* sepals. Genetic and chemical perturbations of BR signaling further demonstrate its role in growth coordination. Crossing *drmy1* with BR mutants or applying brassinolide (a potent brassinosteroid) or brassinazole (a brassinosteroid biosynthesis inhibitor) rescues the sepal elongation defect by differentially altering inner and outer sepal growth. Specifically, in *drmy1* mutants, enhanced BR signaling preferentially promotes inner sepal growth, while reduced BR signaling preferentially decreases outer sepal elongation, thereby restoring more balanced sepal growth.

**Conclusions:**

These results suggest that *DRMY1* is required for proper brassinosteroid-mediated coordination of sepal elongation, ensuring balanced growth between inner and outer sepals during early development.

**Supplementary Information:**

The online version contains supplementary material available at 10.1186/s13059-026-04156-1.

## Background

In developmental biology, robustness refers to the ability of living organisms to produce structures of consistent size and shape within and between individuals despite cell heterogeneity, gene expression stochasticity, and perturbations from the external environment [1, 2]. There are at least three major mechanisms generating robustness during morphogenesis [2, 3]. First, buffering of noise in gene expression, cell growth, and cell division through spatiotemporal averaging allows the overarching patterns of growth to sculpt the organ [4–6]. Second, redundancy in gene regulatory networks allows for recovery from perturbations in an individual gene’s expression [2]. Third, coordination of growth between various parts of a structure allows the organ to reach robust shape [3]. For instance, coordination of cell growth across the medio-lateral axis of the leaf is required for maintaining leaf flatness [7]. Additionally, tissues such as the L1, L2, and L3 layers of the meristem coordinate cell division planes and growth between layers through a combination of hormone and peptide signaling [8, 9]. Growth of the adaxial and abaxial epidermal layers of sepals must also be coordinated to achieve proper curvature [10, 11]. Coordination is also necessary between separate organs on an individual, such as the wings on *Drosophila melanogaster* which must carefully coordinate equally but mirrored growth to achieve symmetric flight [12, 13]. In *Drosophila*, robust inter-organ growth coordination is mediated by a peptide hormone, *DILP8*, which influences transcription factor and signaling pathways to respond to growth perturbations of imaginal disks [14]. In plants, research has shown that cross talk between integument and endosperm helps to coordinate their growth in seeds and the resulting seed size [15]. In Arabidopsis, the cytochrome P450 KLUH/CYP78A5 promotes organ growth via a non-cell autonomous signal that can coordinate growth across the inflorescence [16, 17]. However, little else is known about inter-organ growth coordination in plants.

**Fig. 1:**
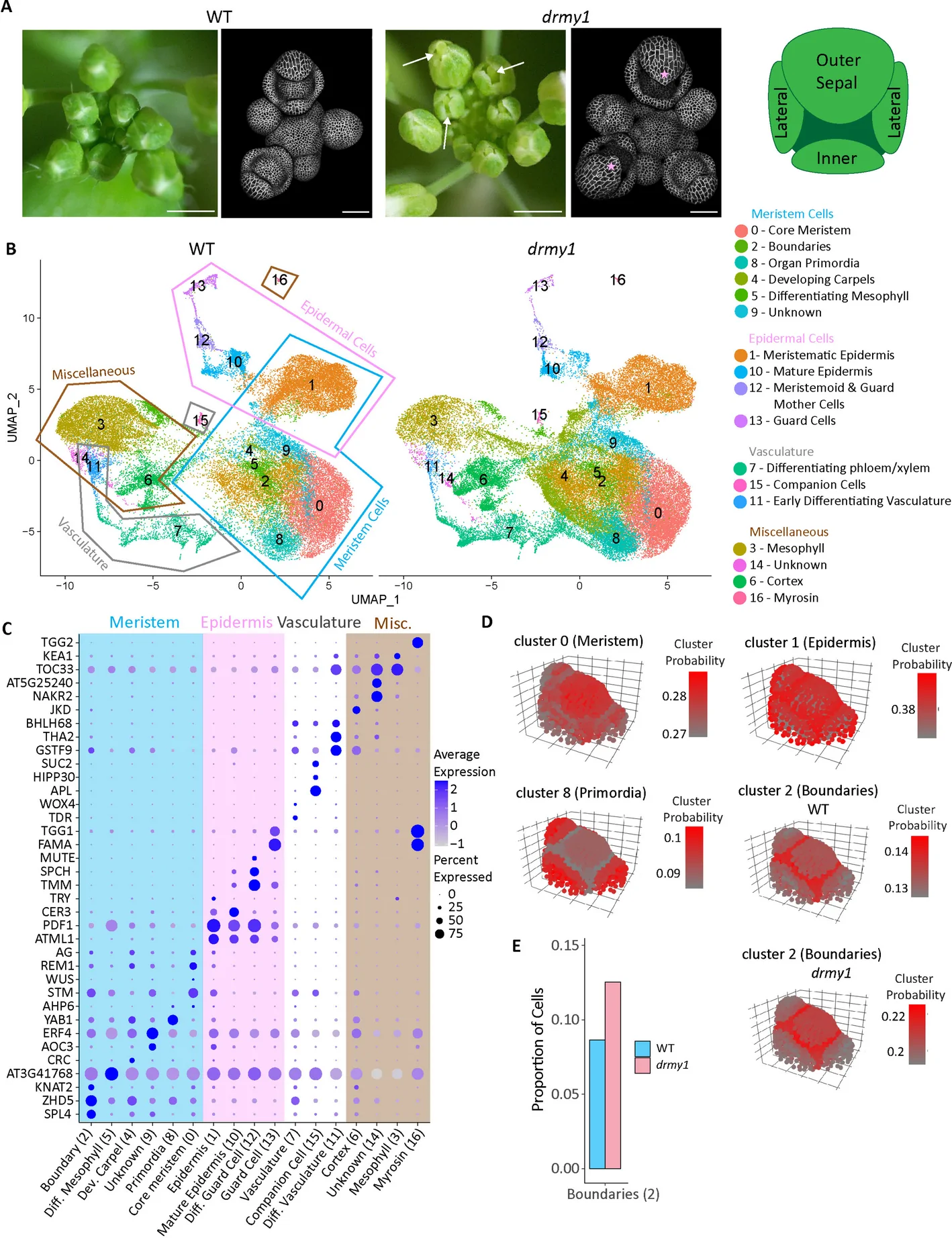
Single cell RNA-seq shows *drmy1* has more boundary cell identity. **A** Wild type (WT) and *drmy1* inflorescences showing flower buds at stages 11–12 (stereomicroscope images) and at stages 0–5 (volume renderings of confocal images). Confocal images were taken of plants expressing the plasma membrane marker *35S::mcitrine-RCI2A*. Note the defects in sepal sizes that prevent stage 11–12 buds from closing (arrows) and the elongated outer sepals in stage 4–5 buds (stars). Scale bars 1 mm (stereomicroscope images) or 50 μm (volume renderings of confocal images). Diagram is used to illustrate where outer sepals (the sepal abaxial to the inflorescence meristem), inner sepals (the sepal adaxial to the inflorescence meristem), and lateral sepals (on either side of the floral meristem, perpendicular to the inflorescence meristem) are found on a typical flower bud. **B** UMAP projection of single cell RNA-seq (scRNA-seq) data with cluster identities labeled. **C** Dotplot of marker genes used to identify cluster identities using SCTransform normalized expression values. **D** Single cell clusters that mapped with high probability onto the floral meristem atlas. Note the higher probabilities for which the boundary cluster maps to the atlas in *drmy1*. All other mappings shown are WT. **E** Proportion of cells assigned to the boundary cluster in WT (0.087) and *drmy1* (0.125). See also: Additional file 1: Fig. S1 and S2

Coordination of growth between organs is extremely important for plant development. For example, flower buds in the model plant organism *Arabidopsis thaliana* are protected by four leaf-like organs called sepals, which includes an outer sepal (the sepal farthest from the inflorescence meristem), an inner sepal (closest to the inflorescence meristem), and two lateral sepals (on either side of the flower bud; Fig. 1A). To properly enclose the flower bud and protect the developing reproductive organs, these four sepals must carefully coordinate the timing of their initiation, rates of elongation, and timing of maturation and growth cessation [4, 18–20]. Thus, sepals provide an ideal model for understanding robustness and growth coordination, which are necessary to achieve the correct size and shape [4, 5, 21, 22].

Previously, we characterized the *development-related myb-like 1* (*drmy1*) mutant, which has uncoordinated initiation and elongation of sepals, leading to sepals with variable size and shape that fail to properly enclose and protect the developing flower bud (Fig. 1A) [18–20]. The *DRMY1* gene encodes a MYB/SANT-domain or protein, suggesting that it may be either a transcription factor or chromatin remodeler. DRMY1 may alter gene expression indirectly through a larger transcriptional or chromatin remodeling complex. DRMY1 transcriptional and translational reporters both indicate that DRMY1 is expressed broadly throughout floral meristems and young flowers and is not restricted to certain cell types [18]. Bulk RNA sequencing (RNA-seq) indicates that in the *drmy1* mutant, translation is reduced and expression of the boundary gene *CUC1* is increased [19, 20]. Consequently, the lower translation rates in *drmy1* reduce the abundance of cytokinin signaling inhibitors ARR7 and AHP6, increasing cytokinin signaling and reducing auxin signaling in young flower buds, which leads to delayed and variable sepal initiation [19]. Intriguingly, changes in auxin and cytokinin escaped detection in bulk RNA-seq experiments, likely because cell-type specific signatures were obscured by the mixed cell population. These limitations can be overcome with single cell RNA-seq (scRNA-seq) and spatial transcriptomics, which preserve cell type and spatial context, respectively, and are important tools in developmental biology [23].

Although variably delayed sepal initiation contributes to the overall loss of robustness in sepal size and shape in *drmy1*, post-initiation growth defects also contribute and are not well understood [18]. In this paper, we investigated how DRMY1 coordinates growth between sepals during the early stages of elongation. We applied comparative single cell RNA-seq (scRNA-seq) and Visium spatial RNA-seq to wild type (WT) and *drmy1* flower buds to identify possible cell type and location specific transcriptomic changes that may impact sepal elongation. We confirmed subtle shifts in auxin and cytokinin signaling in *drmy1* that previously escaped detection by bulk RNA-seq. Our results also revealed miscoordination in brassinosteroid (BR) signaling pathways, which we validated with a BR responsive ratiometric fluorescent reporter. Further, we showed that genetic and chemical modulation of BR signaling differentially affects inner versus outer sepal elongation within the same flower bud. Increasing BR signaling partially rescues sepal elongation defects in *drmy1* by preferentially increasing elongation of the inner sepal, while decreasing BR signaling partially rescues *drmy1* by decreasing overgrowth of the outer sepal. These results highlight a role for DRMY1 in mediating proper levels of brassinosteroid signaling necessary for coordinating the growth between sepals in the *Arabidopsis* flower and provide valuable new insight into the mechanisms driving inter-organ growth coordination in plants.

## Results

### Single cell transcriptomics confirms increased boundary cell identity and suggests decreased translation is cell type specific in young *drmy1* flower buds

In order to capture cell type specific transcriptomic changes contributing to aberrant sepal elongation in young *drmy1* flower buds, we decided to perform single cell RNA sequencing (scRNA-seq). We isolated single cells for this purpose by using a meristem optimized protoplasting protocol [24]. To enrich for flower buds early in sepal development, we introgressed the *drmy1-2* mutation into the *apetala1 cauliflower 35S::APETALA1-GR* (*ap1cal 35S::AP1-GR*) background, which produces large cauliflower-like clusters of densely packed arrested floral meristems. This system has been well established for genetic studies requiring large amounts of floral tissues at similar developmental stages (Additional file 1: Fig. S1a,b) [19, 25–28]. We induced floral initiation with dexamethasone and harvested tissue 108 h later when flower buds spanned from developmental stage 2 (no sepal primordia) to late stage 3 (all sepals initiated and beginning to elongate) [29] (Additional file 1: Fig. S1a). We prepared protoplasts from two independent biological replicates per genotype [*ap1cal 35S::AP1-GR* (“WT”) and *drmy1 ap1cal 35S::AP1-GR* (“*drmy1*”)] and performed 10 × Genomics scRNA-seq. After filtering, we obtained 93,365 high quality cells (mean of 6457 unique molecular identifiers and 2613 features (genes) per cell; Additional file 1: Fig. S1c) and found minimal inter-replicate variability (Additional file 1: Fig. S1d-g), allowing us to combine replicates by genotype for downstream analysis.

Principal component analysis (PCA) initially clustered many cells by cell-cycle phase, even after cell cycle regression was performed (Additional file 1: Fig. S2a), reflecting active cell division in floral meristems. To fully remove the effects of cell cycle on downstream analysis, we excluded genes where > 3% of variance was explained by cell cycle phase (Additional file 2: Table S1). This filtering yielded 17 transcriptionally distinct clusters (Fig. 1B), which we then annotated using established marker genes (Fig. 1C, Additional file 2: Table S2). Broadly, clusters 1, 10, 12, and 13 represent epidermal cells and express epidermal markers such as *ATML1* and *PDF1* (Fig. 1C). Clusters 0, 1, 2, 4, 5, 8, and 9 are largely meristematic cells, expressing markers such as *STM*, *AG*, *WUS*, *CLV1*, *CLV3*, and others (Fig. 1C). Clusters 3 and 5 represent mesophyll cells and have a relatively large proportion of transcripts from chloroplast genes (Fig. 1C, Additional file 1: Fig. S2b). Clusters 7, 11, and 15 express vasculature specific genes such as *APL* and *TDR* (Fig. 1C). *DRMY1* itself has previously been characterized as having low expression broadly across all inflorescence cell types [18]. Likewise, in our single cell dataset *DRMY1* is broadly expressed across all clusters at low levels (Additional file 1: Fig. S2c). For understanding the development of young flower buds, clusters 1 (meristematic epidermis), 10 (mature epidermis), 0 (core meristem), and 8 (organ primordia) are of particular interest since these are the cell types we expect to find most in young flower buds.

Additionally, we aimed to identify boundary cells, given that prior work established overexpression of a boundary identity gene as a key factor in *drmy1* floral development [20]. Particularly, protein and transcriptional reporters for the canonical boundary gene *CUC1* have higher expression levels and expanded expression domains in *drmy1* [20]. However, the *CUC* genes tend to have low overall expression levels, and thus it was difficult to identify boundary cells in our scRNA-seq dataset based on *CUC* expression alone. To help identify these cells, we used a pipeline developed by Neumann et al. [30] that maps scRNA-seq clusters onto a 3-dimensional floral meristem atlas based on shared gene expression patterns. Accuracy of this approach was confirmed by several single cell clusters which mapped with high probability to their expected locations on the floral meristem atlas. This included cluster 0 (core meristem) to the core of the atlas; cluster 1 (meristematic epidermis) to the L1 layer of the atlas; and cluster 8 (primordia) to the sepal primordia (Fig. 1D). Cluster 2 mapped to the boundary between the core meristem and the initiating primordia, indicating probable boundary cells for further analysis (Fig. 1D).

Interestingly, cluster 2 in *drmy1* mapped to the boundary region with higher probability than in WT (0.22 in *drmy1* vs 0.14 in WT) (Fig. 1D). We simultaneously see that cluster 2 (boundaries) tends to represent a higher proportion of the total cell population in *drmy1* (0.125) than in WT (0.087) (Fig. 1E), which may reflect the upregulation of boundary identity in *drmy1*. Other clusters with large differences in cell population proportions include fewer cells in cluster 3 (mesophyll) and more cells in cluster 4 (carpels) in *drmy1* (Additional file 1: Fig. S2d). This is likely because *drmy1 ap1cal 35S::AP1-GR* cauliflower-like clusters have fewer leafy bracts and more flower buds that prematurely develop before dexamethasone induction (Additional file 1: Fig. S1b). However, with only two replicates per genotype, we cannot state with certainty that these trends are significant.

**Fig. 2:**
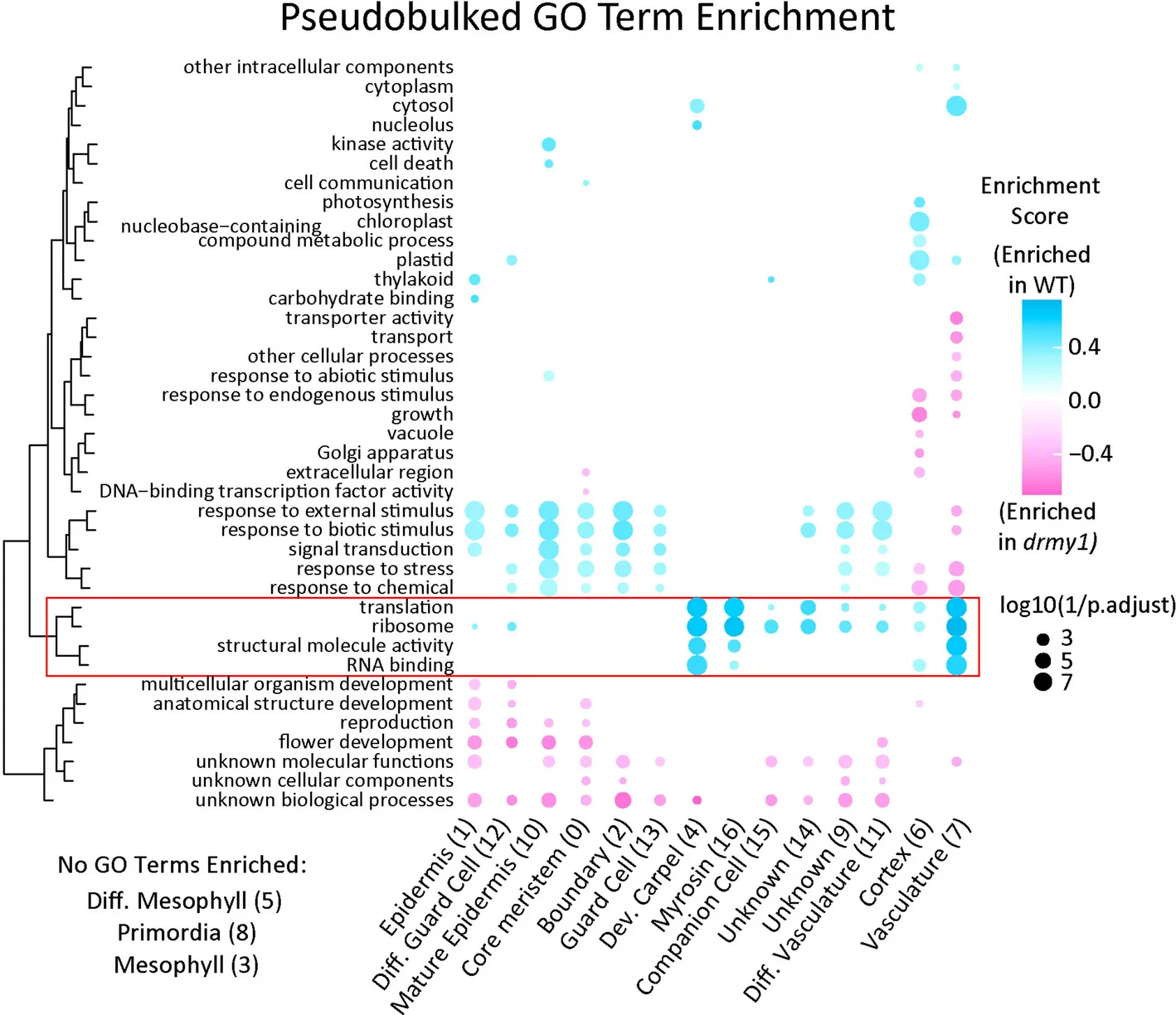
*drmy1* has reduced levels of translation. Gene Ontology Term enrichment of differentially expressed genes in *drmy1*. Blue indicates higher enrichment in WT, pink indicates higher enrichment in *drmy1*. (Additional file 4: Table S4)

To study differential gene expression patterns in *drmy1*, we performed differential gene expression analysis and gene ontology (GO) term enrichment analysis on pseudobulked scRNA-seq data (Additional file 3: Table S3 and Additional file 4: Table S4). Terms related to translation, ribosomes, and RNA binding were greatly enriched in WT compared to *drmy1* (Fig. 2) as seen previously in Kong et al. [19]. Notably however, this enrichment was much lower or absent in clusters 0 (core meristem), 1 (meristematic epidermis), 10 (mature epidermis), 2 (boundaries), 12 and 13 (guard cells) (Fig. 2). This result suggests that despite its non-cell type specific expression, *DRMY1* may be more important for sustaining proper levels of translation in some cell types compared to others. Perhaps some cell types possess unknown mechanisms to buffer against changes in translation rate. In conclusion, our scRNA-seq dataset allowed us to identify key cell types present in developing flower buds while also recapitulating known roles for *DRMY1* in regulating boundary cell identity and translation [19, 20]. Our next step was to test if this dataset would allow us to identify transcriptomic changes in auxin and cytokinin signaling not apparent from bulk RNA-seq, as well as search for additional transcriptomic pathways that *DRMY1* impacts.

### ScRNA-seq recapitulates patterns of decreased auxin signaling, increased cytokinin signaling, and reveals increased brassinosteroid signaling in *drmy1*

**Fig. 3:**
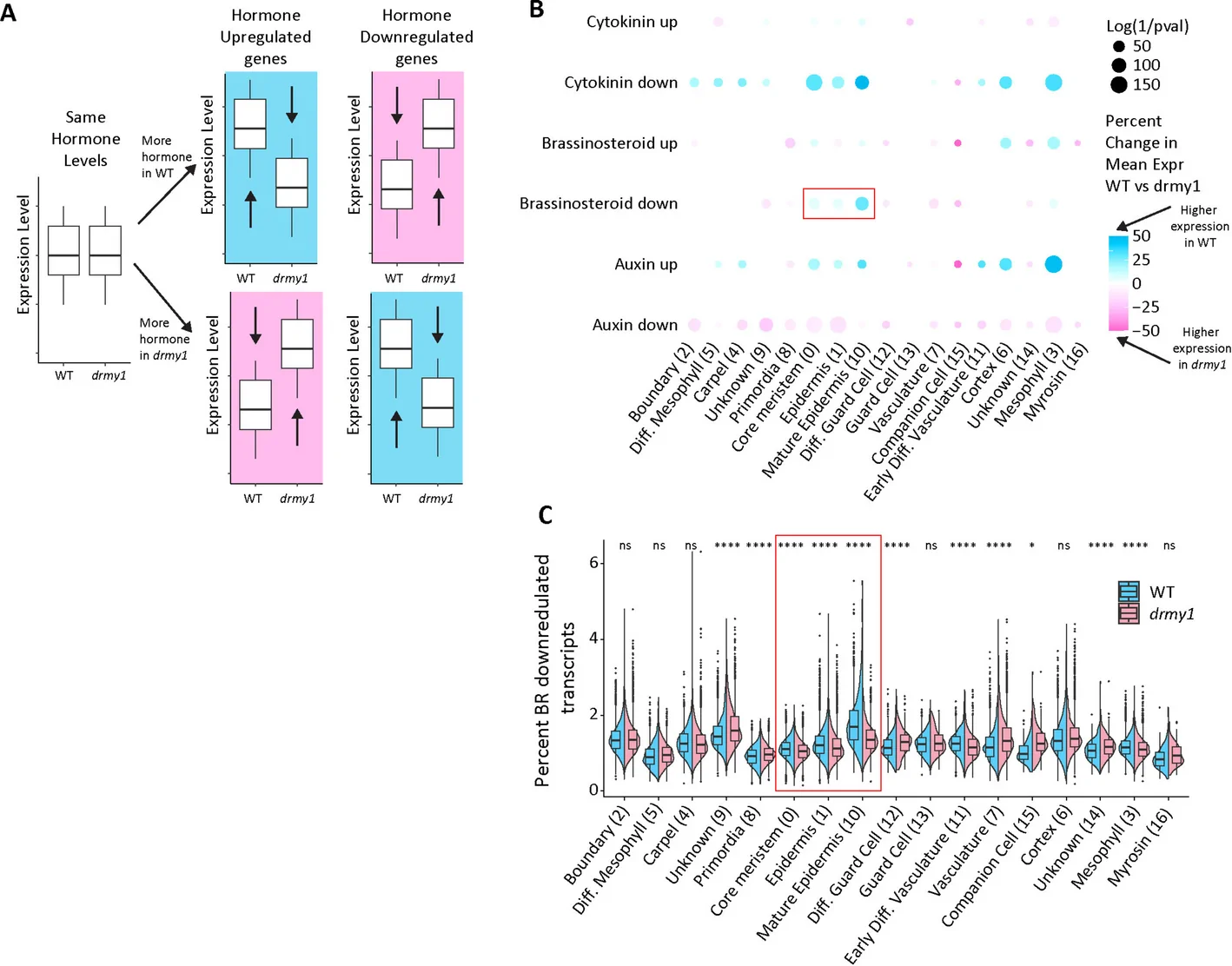
Gene expression trends are consistent with decreased auxin signaling, and increased cytokinin and brassinosteroid signaling in *drmy1*. **A** Schematic of the hypothesized changes in expression levels of hormone up and down-regulated genes under high and low levels of hormone in WT or *drmy1*. Colors correspond to the colors in Fig. 3B, where blue represents higher expression levels in WT and pink represents higher expression levels in *drmy1*. **B** Dotplot showing the differences in expression of genes up and down-regulated by auxin, cytokinin, and brassinosteroids. For each cell, the percentage of transcripts that come from the set of genes up or downregulated by applications of these hormones according to Nemhauser et al. [31] is calculated, then the mean of all the cells in each cluster is calculated for WT and *drmy1*. The size of each dot represents the log of the inverse of the *p*-value between the mean of the two genotypes with a Bonferroni adjusted threshold of *p* < 0.000245. The color of each dot represents the percentage increase or decrease of the mean of *drmy1* compared to WT. Red box indicates changes in BR signaling in clusters of interest in young flower buds (core meristem, epidermis, and mature epidermis). See Additional file 1: Fig. S3 for other hormones, see Additional file 5: Table S5 for lists of up or downregulated genes, Additional file 5: Table S6 for exact *p*-values. **C** Violin plot showing more details of the change in expression patterns from the “Brassinosteroid down” row from panel B. Red box corresponds to the same data boxed in panel B. *p < 0.00294, **p < 0.00059, *** *p* < 0.000059, **** *p* < 0.0000059 Bonferroni adjusted p-value thresholds in Wilcoxon’s rank sum tests compared with WT. See also: Additional file 1: Fig. S3, Additional file 5: Table S5,6

To look for transcriptomic changes in hormone regulated genes, we used a dataset from Nemhauser et al. [31] in which researchers treated Arabidopsis seedlings with various hormones and used microarrays to determine which genes are up and down-regulated and thus are likely downstream transcriptional targets of each hormone (Additional file 5: Table S5). For each cell we calculated what percentage of total transcripts come from genes in each set of up and downregulated genes. We expect higher hormone signaling levels will cause higher expression of hormone upregulated genes and lower expression of downregulated genes, and vice-versa for lower hormone levels (Fig. 3A). For instance, previous work has demonstrated WT has higher levels of auxin signaling than *drmy1* [18, 19], which can be seen in our scRNA-seq data by the prevalence of higher expression (blue dots in Fig. 3B) of auxin upregulated genes and lower expression of auxin downregulated genes (pink dots in Fig. 3B) in WT compared to *drmy1* across most clusters. This suggests that auxin signaling is higher in WT, and it is particularly interesting to note this trend in the meristem (cluster 0) where auxin maxima can eventually give rise to primordia and in mature epidermis (cluster 10) which develops on sepals as they elongate (Fig. 3B). We see the opposite trend with cytokinin regulated genes – particularly with lower expression of cytokinin downregulated genes in *drmy1*, suggesting cytokinin signaling is higher in *drmy1* (Fig. 3B). This trend is especially apparent in clusters 0 (core meristem), 1 (meristematic epidermis), 10 (mature epidermis) (Fig. 3B).

After confirming known changes to auxin and cytokinin signaling, we performed the same analyses on other hormones as well, especially looking for changes in the core meristem, epidermal, and boundary clusters. Genes involved in brassinosteroid (BR) signaling stood out, especially in the core meristem and epidermal clusters where BR downregulated genes have lower expression in *drmy1* (Fig. 3B, C), which suggests possible BR upregulation in *drmy1*. We also looked at individual genes associated with brassinosteroid GO terms. Notably, BR biosynthesis gene *DWF1* is upregulated in *drmy1* (Additional file 1: Fig. S3a). In addition, *DWF4* BR biosynthesis gene is downregulated in *drmy1*, which further suggests higher BR signaling in *drmy1* since *DWF4* is downregulated by brassinosteroids in a negative feedback loop with BR levels [32] (Additional file 1: Fig. S3a). *BSK2*, a core component of the BR signaling pathway, is also upregulated in *drmy1* (Additional file 1: Fig. S3a). We performed similar analyses with other hormones such as abscisic acid, jasmonic acid, and gibberellic acid, and while there were notable changes in their expression patterns in several clusters, the overall trends were less clear in our clusters of interest, thus we focused primarily on BR going forward (Additional file 1: Fig. S3b). Additionally, using Gene Set Enrichment Analysis (GSEA) we were able to confirm that the set of BR upregulated genes are significantly enriched in many *drmy1* clusters (Additional file 1: Fig. S3c)**.** Curiously, the enrichment does not appear to be as cell-type specific as suggested by the analysis of transcript levels. These results suggest that BR signaling may be higher in *drmy1* than wild type, and therefore *DRMY1* may be important for regulating BR signaling.

### Visium spatial RNA-seq corroborates scRNA-seq trends with brassinosteroids and boundaries

**Fig. 4:**
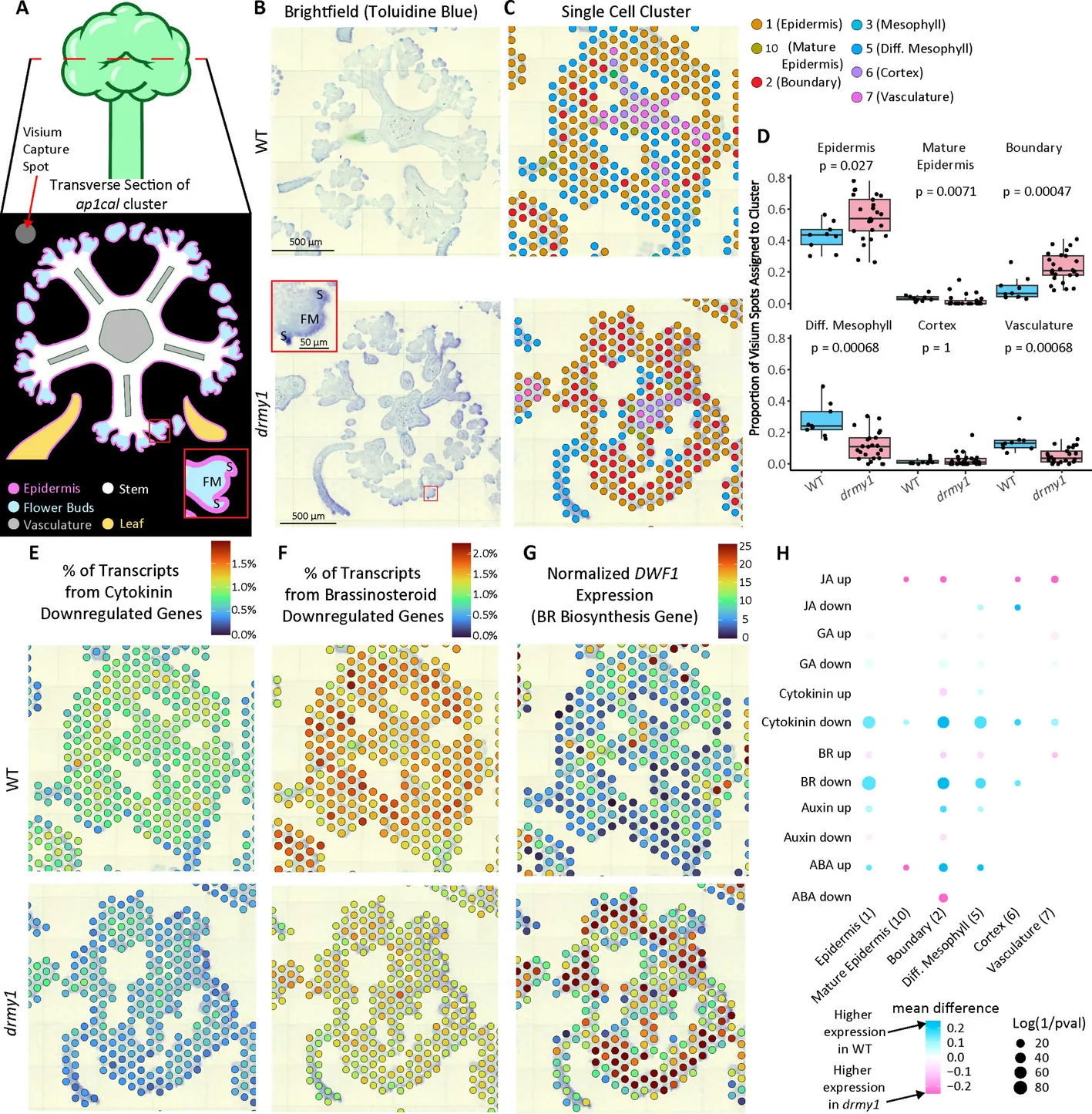
Spatial RNA-seq further suggests boundary identity, cytokinin signaling, and brassinosteroid signaling are increased in *drmy1*. **A** Cartoon of a typical transverse section of an *ap1cal 35S::AP1-GR* cauliflower-like cluster, showing the developing flower buds with initiating sepals on the outer ring. Inset shows an enlarged depiction of a floral meristem (FM) and its two sepals (S). **B** Representative brightfield images of transverse sections of WT and *drmy1* in the *ap1cal 35S::AP1-GR* background. Inset shows an enlarged image of a floral meristem (FM) and its two sepals (S). *n* = 9 Total WT replicate cauliflower-like clusters (after removal of one WT capture area with abnormally high mitochondrial read levels), *n* = 25 *drmy1* replicate cauliflower-like clusters (See also Additional file 1: Fig. S4,5). **C** An overlay of which single cell cluster maps with the highest prediction score to each Visium spot on the same tissue shown in panel (**B**). See Additional file 1: Fig. S4 for a more detailed view of prediction scores from each single cell cluster. **D** Boxplots showing the proportion of Visium spots in each cauliflower-like cluster that map best to each scRNA-seq cluster based on Seurat deconvolution prediction scores. scRNA-seq clusters represented by < 10 Visium spots in either genotype were omitted. *p* = 0.027 (Epidermis), *p* = 0.0071 (Mature Epidermis), *p* = 0.00047 (Boundaries), *p* = 0.00068 (Differentiating Mesophyll), *p* = 1 (Cortex), *p* = 0.00068 (Vasculature) in Wilcoxon’s rank sum test. *n* = 9 WT samples and n = 25 *drmy1* samples. **E**, **F** Overlays that show what percentage of all the transcripts captured at a given spot come from cytokinin downregulated genes (**E**) or brassinosteroid downregulated genes (**F**). **G** SCT normalized expression values of *DWF1*, a brassinosteroid biosynthesis gene. **H** Dotplot showing the differences in expression of genes up and down-regulated by various hormones. For each Visium spot, the percentage of transcripts that come from the set of genes up or downregulated by applications of these hormones according to Nemhauser et al. [31] is calculated, then the mean of all the Visium spots that are predicted to map best to a given single cell cluster is calculated for WT and *drmy1*. The size of each dot represents the log of the inverse of the *p*-value between the mean of the two genotypes with a Bonferroni adjusted threshold of *p* < 0.00069. The color of each dot represents the percentage increase or decrease of the mean of *drmy1* compared to WT. See Additional file 5: Table S6 for exact *p*-values. Scale bars 500 μm. See also: Additional file 1: Fig. S4,5, Additional file 5: Table S6

While single cell RNA-seq offers excellent resolution, it does not preserve spatial information. Therefore, to understand the spatial patterns of gene expression changes in *drmy1*, we applied Visium Spatial RNA-seq to 10 μm sections of floral meristem clusters from WT and *drmy1* in *ap1cal 35S::AP1-GR* backgrounds 108 h post induction, when numerous flower buds on the cauliflower-like head are starting to initiate sepals. These transverse slices allow for easy visual identification of many common cell types and anatomical structures (Fig. 4A). Although the Visium capture spots were slightly too large (55 μm diameter spots placed 100 μm apart) to compare gene expression profiles from different regions of individual flower buds (about 100 μm in diameter), we were still able to use Seurat Deconvolution to predict the cell type composition of the spots based on our single cell clusters (Fig. 4B-D, Additional file 1: Fig. S4b, S5). In agreement with our scRNA-seq results, boundary cell identity (cluster 2) had the highest prediction score in a higher proportion of Visium spots in *drmy1* than in WT (*p* = 0.00047) (Fig. 4C, D). Fitting with the morphology, the boundary identity (cluster 2) maps best to spots just interior to epidermis-mapping (cluster 1) spots on the edges of floral meristem clusters (Fig. 4C and Additional file 1: Fig. S4b). In addition to prediction scores, we can assess boundary identity by calculating module scores in Seurat using the top 100 conserved markers from the scRNA-seq boundary cluster as our module (Additional file 5: Table S7). We find that module scores positively correlate with prediction scores for the boundary cluster (Additional file 1: Fig. S4c) (p < 2.2 × 10^–16^), and that both prediction scores (p < 2.2 × 10^–16^) and module scores (p = 2.3 × 10^–14^) are significantly higher in *drmy1* compared to WT (Additional file 1: Fig. S4d,e). Both methods thus suggest that our spatial RNA-seq dataset recapitulates previously reported increases in boundary identity in *drmy1* [20], validating that despite the lower resolution of the spatial dataset compared to the sc-RNAseq dataset, we can still identify changes in cell type specific expression profiles.

To clarify the spatial distribution of hormone signaling, we summarized the difference in the composition of sets of genes up and downregulated by various hormones between WT and *drmy1* (Fig. 4E-H). We find that cytokinin and BR downregulated genes have much higher expression in WT, especially in spots whose prediction scores are highest for the epidermis, boundary, and mesophyll identities (Fig. 4H). This further supports the hypothesis that cytokinins and BR are upregulated in *drmy1*, especially in spots on and nearby to young flower buds (Fig. 4E, F). Similarly, key BR genes such as the BR biosynthesis gene *DWF1* are highly upregulated in *drmy1* spots on and near the flower buds (Fig. 4G). Together, these results indicate that *DRMY1* may be important for maintaining proper brassinosteroid signaling levels, so next we tested the location and role of BR signaling in sepal development.

### A ratiometric BES1 reporter confirms brassinosteroid signaling is upregulated in *drmy1*

**Fig. 5:**
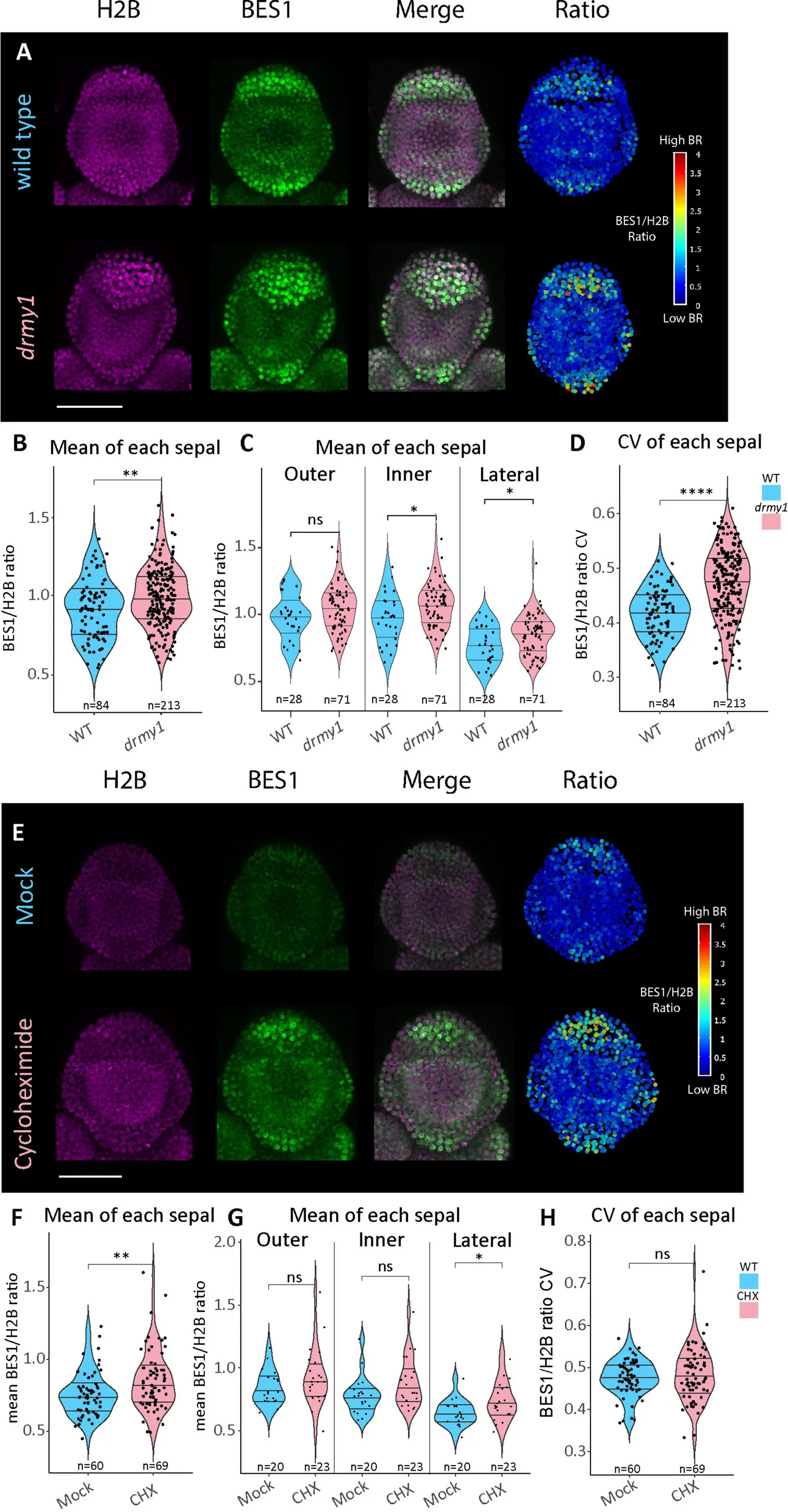
A BES1 ratiometric reporter confirms brassinosteroid signaling is upregulated in *drmy1*. **A** WT and *drmy1* expressing *pUBQ10::H2B-mcherry* (magenta)/*pUBQ10::BES1-ypet* (green), both channels merged, and a heat map of the ratio of the BES1 signal divided by the H2B signal in each nucleus. **B** Quantification of the mean BES1/H2B signal ratio for each sepal, *p* = 0.0041. Sample sizes for panels B through D are higher in *drmy1* to better capture increased variability in BES1/H2B ratios and because *drmy1* inflorescence meristems tend to have more flower buds than WT as previously reported [18]. See also Additional file 1: Fig. S6. **C** Quantification from (**B**) separated by sepal position, *p* = 0.15 (outer), *p* = 0.025 (inner), *p* = 0.048 (lateral). **D** CV of BES1/H2B ratio in each sepal, *p* = 6.5 × 10^–12^. **E** Day 6 mock and 2 μM cycloheximide treated buds expressing the BES1/H2B ratiometric reporter. **F** Quantification of the mean BES1/H2B signal ratio for each sepal, *p* = 0.0049. **G** Quantification from (**F**) separated by sepal position, *p* = 0.18 (outer), *p* = 0.059 (inner), *p* = 0.03 (lateral). **H** CV of BES/H2B ratio in each sepal, *p* = 0.62. Lines in violin plots represent the 25th, 50th, and 75th quantiles. For each graph, n is listed below the violin plot. *p < 0.05, **p < 0.005, *** *p* < 0.0005, **** *p* < 0.00005 in Wilcoxon’s rank sum tests. Scale bars 50 μm

To confirm that BR signaling is upregulated in *drmy1*, we introgressed the ratiometric BRI1-EMS-SUPPRESSOR1 (BES1) reporter (*pUBQ10::BES1-ypet/pUBQ10::H2B-mCherry*) [33] into *drmy1*. Compared to WT, *drmy1* sepals had significantly higher mean BES1/H2B ratios (*p* = 0.0041) (Fig. 5A, B and Additional file 1: Fig. S6). When broken down by sepal position, we find that BR signaling was significantly higher in *drmy1* inner and lateral sepals, while in outer sepals the difference is insignificant but trends higher (outer; *p* = 0.15, inner; *p* = 0.025, lateral; *p* = 0.048) (Fig. 5C). In our single cell and spatial RNA-seq data, we could not distinguish between inner, outer, and lateral sepals, so the fluorescent reporter added new information. In addition, the coefficient of variance (CV) of BES1/H2B ratios within individual sepals is significantly higher in *drmy1* (*p* = 6.5 × 10^–12^) (Fig. 5D and Additional file 1: Fig. S6), which is consistent with *drmy1* having increased variability in auxin signaling as well [20].

Previous work has shown that treating WT buds with the translation inhibitor cycloheximide (CHX) can recreate several phenotypes seen in *drmy1,* such as sepal growth defects and changes to auxin and cytokinin signaling [19]. Therefore, we hypothesized that we should be able to recreate increases in brassinosteroid signaling with CHX as well. As anticipated, treating WT plants with 2 μM CHX for 6 days significantly increased BES1/H2B ratios of sepals (*p* = 0.0049) (Fig. 5E, F). This result was only significant for lateral sepals, though trended higher in outer and inner sepals (outer; *p* = 0.18, inner; *p* = 0.059, lateral; *p* = 0.03) (Fig. 5G). However, CHX did not recapitulate the increase in CV seen in *drmy1* (Fig. 5H), suggesting that the increased variability in BR signaling in *drmy1* is not a direct consequence of the decreased translation. Together, these results confirm that brassinosteroid signaling is increased in *drmy1* likely due to the decrease in protein translation. This effect may be indirect as suggested by CHX treatment results.

### Altering brassinosteroid levels differentially affects elongation of the inner and outer sepals

**Fig. 6:**
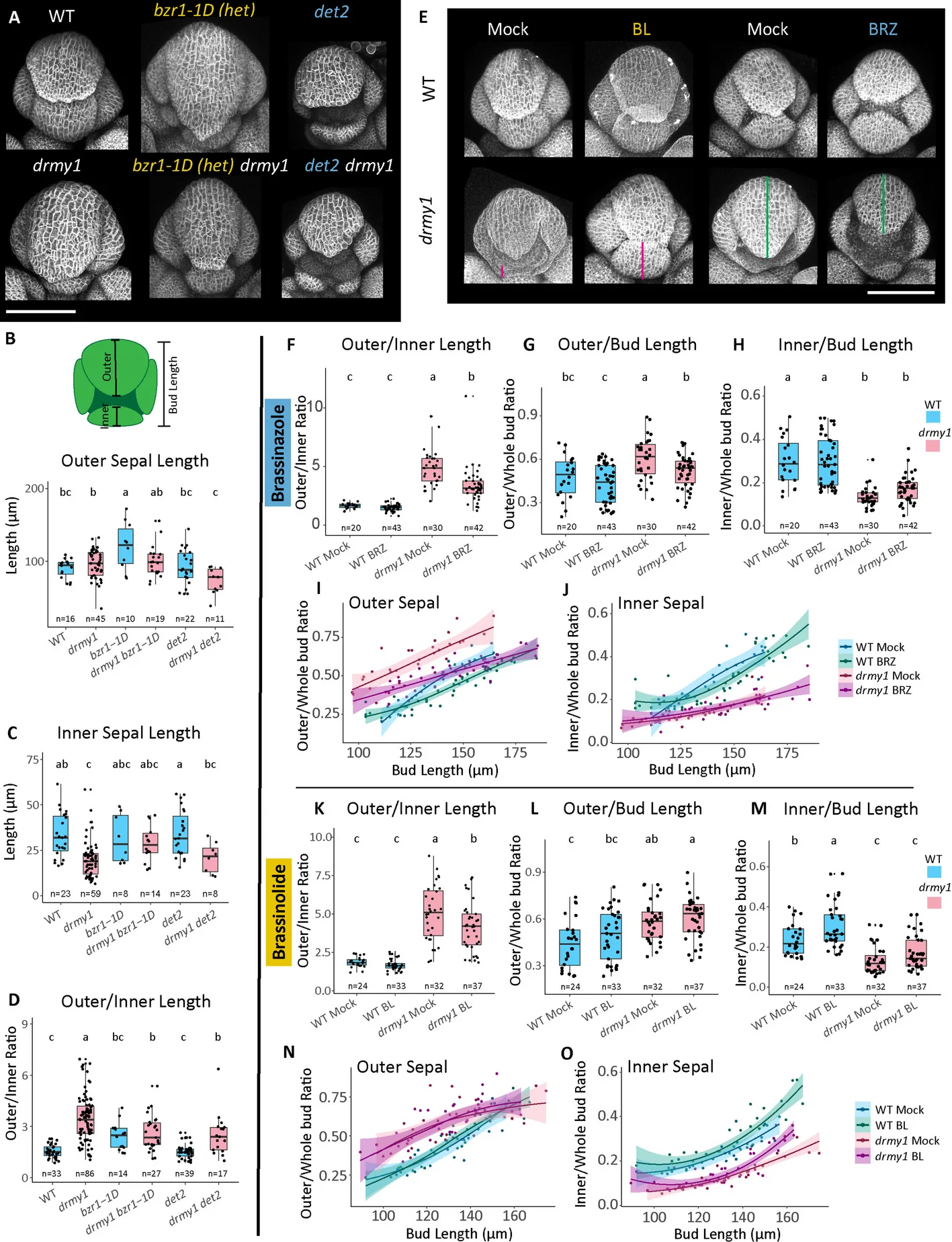
Sepal growth defects are partially rescued in *drmy1* by decreasing brassinosteroid signaling to reduce outer sepal overgrowth, or by increasing brassinosteroid signaling to increase inner sepal growth. **A** Confocal images of single brassinosteroid mutants *bzr1-1D* and *det2*, as well as double mutants with *drmy1* expressing the membrane marker *35S::RCI2A-mCitrine*. Heterozygous *bzr1-1D* lines are shown as representative images to demonstrate that the gain-of-function phenotype is visible with just a single copy of *bzr1-1D*. Homozygotes had visually similar phenotypes. **B** – **D** Quantification of the lengths of the outer sepals of stage 5–7 flower buds (**B**), inner sepals of stage 3–5 flower buds (**C**), and the ratio between the length of the outer sepal divided by the length of the inner sepal in all flower buds (**D**) in single and double mutants. *bzr1-1D* lines include both heterozygotes and homozygotes. **E** Confocal images of WT and *drmy1* flower buds treated for 6 days with either 50 uM brassinazole (BRZ), a brassinosteroid biosynthesis inhibitor, 400 nM brassinolide (BL), or mock treatments. Buds are either expressing the membrane marker *35S::RCI2A-mCitrine* or are stained with 0.1 mg/mL propidium iodide. Note magenta bars indicating rescued growth of the inner sepal in BL treated *drmy1* buds, and the green bars indicating reduced overgrowth of outer sepals in BRZ treated *drmy1* buds. **F**–**H** Quantification of the ratio between the lengths of the outer and inner sepals (**F**), the ratio between the lengths of the outer sepal and the entire bud (**G**), and the ratio between the lengths of the inner sepal and the entire bud (**H**) after mock or BRZ treatment. The length of the entire bud is an indication of the developmental stage since the bud increases size as it grows and allows us to normalize for bud stage, which ranges from stages 3–7. **I**, **J** Relationship between the length of the entire bud and the ratio between the lengths of the outer sepal (**I**) or the inner sepal (**J**) after mock or BRZ treatment. Lines represent the Loess regression fit of the data and shaded areas represent the 95% confidence interval. **K**-**M** Quantification of the ratio between the lengths of the outer and inner sepals (**K**), the ratio between the lengths of the outer sepal and the entire bud (**L**), and the ratio between the lengths of the inner sepal and the entire bud (**M**) after mock or BL treatment. The length of the entire bud is an indication of the developmental stage since the bud increases size as it grows and allows us to normalize for bud stage, which ranges from stages 3–7. **N**, **O** Relationship between the length of the entire bud and the ratio between the lengths of the outer sepal (**N**) or the inner sepal (**O**) after mock or BL treatment. Lines represent the Loess regression fit of the data and shaded areas represent the 95% confidence interval. Different lowercase letters indicate significant differences based on one-way analysis of variance (ANOVA) with Tukey's honest significant difference test (*P* < 0.05; statistical details in Additional file 5: Table S8). n is listed on the graph for each line and represents the number of flower buds measured. The boxes extend from the lower to upper quartile values of the data, with a line at the median. The whiskers extend to 1.5 of the interquartile range. Scale bars 100 μm. See also: Additional file 1: Fig. S7, Additional file 5: Table S8

Because *drmy1* has increased BR signaling, we investigated BR mutants for sepal growth defects. We noticed that the dominant *brassinazole-resistant1-1D* (*bzr1-1D*) mutant, which has higher levels of BR signaling due to a stabilized BR responsive transcription factor, has elongated outer sepals compared to WT, reminiscent of the elongated outer sepals of *drmy1* (Fig. 6A, B). Interestingly, *bzr1-1D* inner sepals are not longer than WT inner sepals (Fig. 6C). This results in a slightly larger discrepancy between the lengths of the inner and outer sepals in *bzr1-1D* compared to WT, which we quantified by taking the ratio of the lengths of the outer divided by the inner sepals (Fig. 6D). However, the outer/inner ratio was not quite as severe in *bzr1-1D* as in *drmy1*, suggesting that increased BR signaling accounts for some, but not all, of the size discrepancy between the outer and inner sepals in *drmy1* (Fig. 6D).

We next wondered if reduced BR levels would alter the relative elongation of the outer and inner sepals flower buds. For this we used the *de-etiolated2* (*det2*) mutant, which has greatly reduced levels of brassinolide biosynthesis. Interestingly, *det2* does not significantly alter the lengths of the inner or outer sepals compared to WT, and thus does not affect the outer/inner sepal ratio (Fig. 6A-D). This may suggest that WT sepals are relatively robust to drops in brassinosteroid levels, as perhaps other growth factors such as auxin are able to compensate.

Next we wanted to know if crossing *drmy1* with these BR mutants could rescue or worsen the sepal elongation defects. We hypothesized that further increasing BR signaling by crossing *drmy1* with *bzr1-1D* would accentuate outer sepal overgrowth, but were surprised to find that outer sepal length remained unchanged in *drmy1 bzr1-1D* double mutant compared to *drmy1* alone (Fig. 6A, B). This suggests the outer sepal growth induced by BR signaling may already be saturated, as higher endogenous BR levels can lead to weaker responses to additional BR compared to plants with lower BR levels [34]. Instead, we noticed that the inner sepal length trended longer in the double mutant although failed to reach significance. (Fig. 6A, C), leading to a partial rescue of the outer/inner sepal ratio, suggesting that further increasing BR signaling may promote elongation of the inner sepal (Fig. 6D).

Since BR signaling is upregulated in *drmy1*, we hypothesized that crossing *det2* with *drmy1* may rescue the sepal primordium size coordination defect in *drmy1* by reducing outer sepal overgrowth. Compared with *drmy1*, the *drmy1 det2* double mutant has reduced outer sepal length while inner sepal length remains unchanged (Fig. 6A-C). As a result, the outer/inner sepal ratio is partially rescued in *drmy1 det2* compared to *drmy1* (Fig. 6D). These results suggest that decreasing BR signaling and biosynthesis levels can partially rescue the *drmy1* sepal primordium size coordination defect by limiting the overgrowth of the outer sepal.

To verify the trends from our mutant analysis, we altered BR signaling through chemical treatments as well. We achieved this by applying 400 nM brassinolide (BL), the most bioactive brassinosteroid, or 50 μM brassinazole (BRZ), an inhibitor of DWF4, a key enzyme in the BR biosynthesis pathway (Fig. 5E-O). Dipping inflorescences in solutions of these chemicals daily for 6 days clearly altered tissue growth patterns in expected ways, such as increasing (in the case of BL) or decreasing (in the case of BRZ) pedicel and silique lengths (Additional file 1: Fig. S7a). As predicted from our mutant experiments, both BL and BRZ applications lead to a partial rescue of sepal primordium size coordination as represented by the outer/inner sepal ratios in *drmy1* (Fig. 6E, F, K). Conversely, WT buds are robust to changes in their outer/inner sepal ratios (Fig. 6E, F, K). As anticipated, BRZ significantly decreases the length of the outer sepal normalized to the length of the whole bud in *drmy1* (p = 0.0094) (Fig. 6G, I). BRZ has no obvious effect on inner sepal relative length in *drmy1* (Fig. 6H, J). Altogether, these results corroborate the idea that decreasing BR levels can reduce outer sepal overgrowth in *drmy1* without affecting inner sepal growth.

Conversely, BL did not lead to any significant changes in outer sepal size normalized to the whole bud in *drmy1* (Fig. 6L-N). Instead, BL treatments increased the size of the inner sepal normalized to the whole bud in both WT and *drmy1*, although failed to reach significance in the latter (Fig. 6M). This effect was more pronounced at later stages of growth, when buds are larger, possibly because later stages have had more time to accumulate more growth (Fig. 6O).

The trend towards increasing *drmy1* inner sepal growth under BL treatment mirrors the increased length of the *drmy1* inner sepal when crossed with *bzr1-1D*. Likewise, the decrease of *drmy1* outer sepal growth under BRZ treatment mirrors the decreased length of the *drmy1* outer sepal when crossed with *det2*. Surprisingly, BL did not seem to increase outer sepal length in WT, while the *bzr1-1D* had longer sepals than WT. This may be because WT is more robust than *drmy1* and may require longer or more constitutive treatments to see an effect similar to *bzr1-1D*, which is constitutively active.

From prior work, we know that a large contributor to the defects in *drmy1* sepal development is the variable and delayed sepal initiation caused by decreased auxin signaling and increased CUC1 expression [18, 20]. To ensure the partial rescue from BRZ and BL treatments is a result of changes to BR mediated elongation and not simply due to rescuing sepal initiation, we imaged treated *drmy1* plants every 6 h and recorded when sepals initiated. In doing so, we found that the treatments did not rescue sepal initiation timing in *drmy1* (Additional file 1: Fig. S7b). Furthermore, to rule out cross talk between BR and auxin or CUC1, we applied these treatments to plants expressing *pCUC1::3xVenus-N7* and to *drmy1* plants expressing the auxin reporter *DR5::Venus-N7*. We found no obvious changes to the expression intensity or pattern of either reporter (Additional file 1: Fig. S7c,d). These results confirm that increasing BR levels partially rescues *drmy1* sepal primordium elongation defects by increasing the growth of the inner sepal, while decreasing BR levels partially rescues by decreasing the overgrowth of the outer sepal, and that these effects are independent of the defects in sepal initiation caused by auxin and CUC1 (Additional file 1: Fig. S7e). This confirms the role *DRMY1* plays in brassinosteroid mediated control over sepal elongation is independent of the role *DRMY1* plays in auxin and CUC1 mediated sepal initiation.

### Altering brassinosteroid levels alters relative sepal sizes by changing outer and inner sepal growth rates

**Fig. 7:**
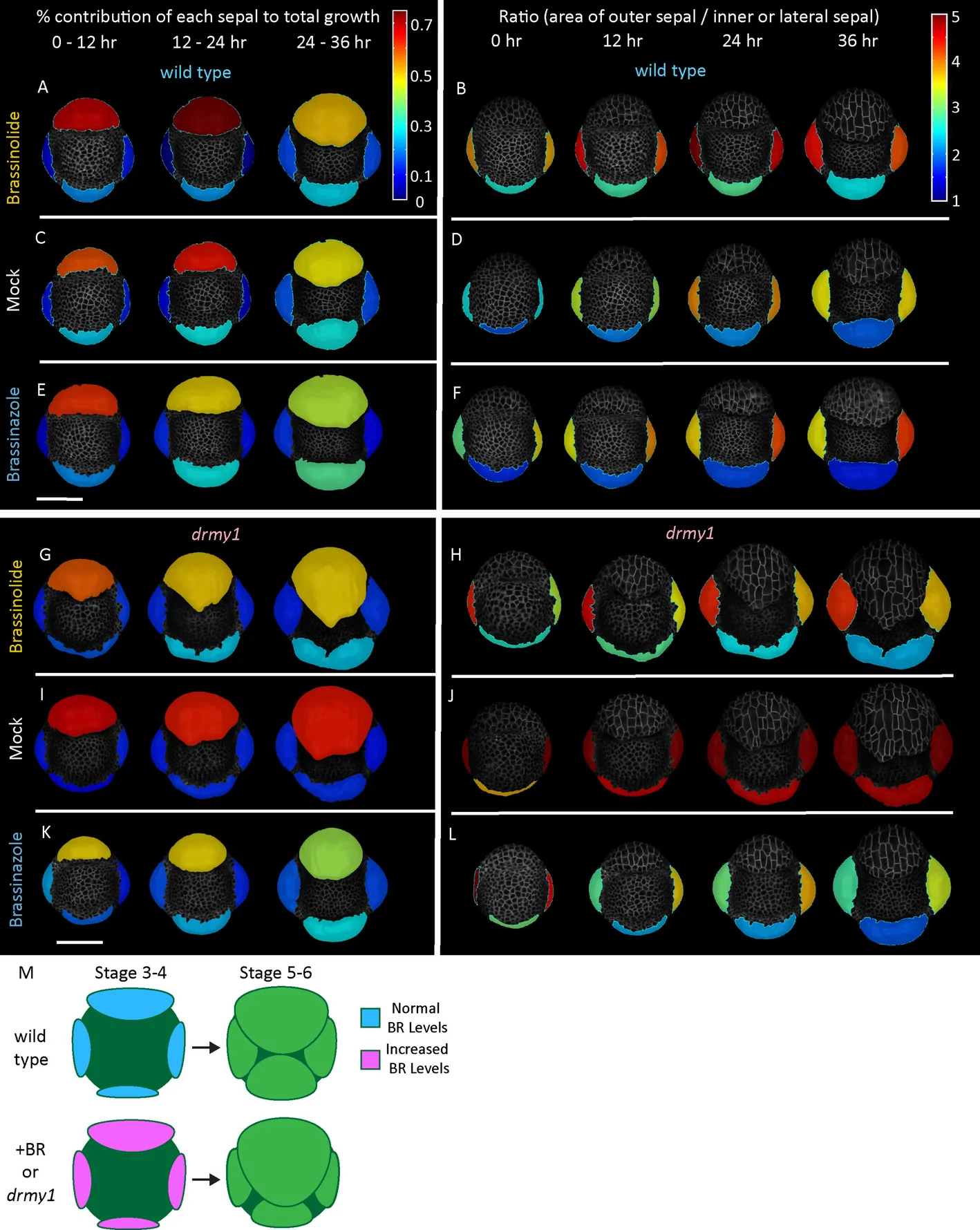
Brassinazole reduces outer/inner sepal ratios by reducing outer sepal growth, brassinolide by increasing inner sepal growth. **A**, **C** and **E** Proportion of total sepal growth contributed by each sepal in WT buds treated with 400 nM brassinolide (BL) (**A**), mock (**C**), or 50 μM brassinazole (BRZ) (**E**) at 12 h time intervals. **B**, **D**, and **F** Corresponding ratio between the area of the outer sepal to the area of the inner and each lateral sepal under BL (**B**), mock (**D**), and BRZ (**F**) treatments in WT buds. **G**, **I** and **K** Proportion of total sepal growth contributed by each sepal in *drmy1* buds treated with 400 nM brassinolide (BL) (**G**), mock (**I**), or 50 uM brassinazole (BRZ) (K) at 12 h time intervals. **H**, **J** and **L** Corresponding ratio between the area of the outer sepal to the area of the inner and each lateral sepal under BL (**H**), mock (**J**), and BRZ (**L**) treatments in *drmy1* buds. **M** Model representing the role of increased BR signaling or the *drmy1* mutation on the growth of the *Arabidopsis* flower buds. Scale bars 50 μm. See also: Additional file 1: Fig. S8, S9, S10

To better understand the impact of altering BR levels on growth patterns of the different sepals, we live imaged WT buds with BL (Fig. 7A, B), mock (Fig. 7C, D), and BRZ (Fig. 7E, F) treatments. We normalized for differences in overall growth rates for buds within and between treatments by calculating the total change in area of all sepals, and then determining the proportion of the total change in area that comes from each sepal. In mock treated WT buds, the outer sepal accounts for a slight majority of growth between each timepoint (typically 50–60%), while the inner sepal accounts for less (20–30%) of the growth, leading to relatively small outer/inner sepal ratios (Fig. 7C, D). Treatment with BL increases the relative growth of the outer sepal, particularly at earlier timepoints (60–70%), leading to slightly larger outer/inner sepal ratios (Fig. 7A, B). Meanwhile, BRZ slightly decreases the growth contributions of the outer sepal, particularly at later timepoints (40–50%), although the effect is small enough that the outer/inner sepal ratio is largely unchanged (Fig. 7E, F). Additional replicates show similar trends (Additional file 1: Fig. S8). Also, segmenting every cell in the sepal shows that these differences in growth rates are driven largely by cells in the sepal tips, which are the fastest growing cells at these early stages (Additional file 1: Fig. S9a-f). These results recapitulate trends seen in our single timepoint images, which demonstrate that BL can increase outer sepal growth and BRZ can decrease outer sepal growth in WT, but that growth remains mostly robust.

We also live imaged *drmy1* buds with BL (Fig. 7G, H), mock (Fig. 7I, J), and BRZ (Fig. 7K, L) treatments. In contrast to the mock WT buds, outer sepals from mock treated *drmy1* buds account for a much larger proportion of sepal growth (> 70%), leading to very high outer/inner sepal ratios (Fig. 7I, J). Applying BL reduces outer sepal growth percentage to near WT levels (50–60%) and increases inner sepal growth percentage (20–30%), greatly reducing the outer/inner sepal ratios (Fig. 7G, H). Furthermore, absolute growth amounts support that the amount of growth in the outer sepal is similar between BL and mock, but inner sepal growth has increased (Additional file 1: Fig. S9g-l). BRZ treated buds have greatly reduced outer sepal growth contributions (40–50%) (Fig. 7K, L), while absolute growth rates confirm that the inner sepal remains relatively unaffected (Additional file 1: Fig. S9l). Additional replicates show similar trends, and intriguingly, the severity of the growth defect as visualized by outer/inner sepal ratio is worse in samples where outer sepal growth contributions are higher (Additional file 1: Fig. S10). Our results confirm that increasing BR levels in *drmy1* partially rescues sepal elongation defects by increasing inner sepal growth and not altering outer sepal growth, while decreasing BR levels partially rescues by decreasing outer sepal growth rates without altering inner sepal growth rates (Fig. 7M).

## Discussion

Growth coordination is an essential facet of developmental robustness. By profiling transcriptomic changes at the single cell level in wild type versus *drmy1* mutants, we were able to show that brassinosteroids, which are key regulators of cellular growth and elongation, play an important role in growth coordination of the four elongating sepals during the early development of the flower bud. BR signaling is upregulated in *drmy1* during early sepal elongation, causing a lack of proper coordination between the elongation of the outer and inner sepals, hindering robust sepal development. We use BR mutants to demonstrate the upregulation of BR signaling can lead to overgrowth of the outer sepal (Fig. 6B). This is similar to the overgrowth defect seen in *drmy1*, which demonstrates that proper BR signaling levels are required for coordination of outer and inner sepal growth (Fig. 7M). We also use brassinolide and brassinazole treatments to show that increasing BR signaling in *drmy1* can partially rescue the sepal size discrepancies by increasing growth of the inner sepal, while decreasing BR signaling partially rescues by decreasing overgrowth of the outer sepal (Additional file 1: Fig. S7e). Thus, the differential effects of BR signaling on different sepals may be essential for the proper coordination of sepal elongation during early flower development to rapidly enclose and protect the developing flower bud. 

The differential effects of BR signaling on outer and inner sepal primordium elongation seen here are not entirely surprising given that previous research has shown BR can have different effects on different populations of cells or tissues [35]. For example, in rice leaves BR preferentially inhibits cell proliferation on the abaxial side of the lamina joint, causing changes in leaf erectness [36].

Previous studies of BR in the shoot apical meristem have shown that downregulation of BR in boundary cells is important for proper *CUC* gene expression and boundary formation [37, 38]. It is surprising, then, that despite a clear upregulation of boundary gene identity in *drmy1* (Fig. 1E; Fig. 4D) [20], that BR signaling is still overall higher in *drmy1* (Fig. 5A, B). However, a closer look at the boundary cell type in our single cell data shows that BRs are in fact not upregulated in the boundaries (cluster 2) (Fig. 3). Rather, both spatial and single cell datasets suggest BR signaling is higher in *drmy1* epidermal cells compared to WT, thus we hypothesize that the differences in sepal outgrowth between WT and *drmy1* may be driven by increased signaling in the epidermis of sepal primordia. This potentially reflects previous work that suggests BR may act predominantly through the epidermis to affect growth [39–41]. However, the relative importance of the epidermis is still controversial, as other research suggests low levels of receptor expression in other cell layers are essential for proper BR mediated growth [42].

Intriguingly, the BR treatments in *drmy1* do not appear to alter auxin signaling mediated sepal initiation, suggesting that the partial rescue of sepal elongation is not a direct consequence of BR – auxin crosstalk. However, the interplay between these hormones is often very complex and changes depending on the circumstances and location [43, 44]. In some instances, such as root apices, BR and auxin have opposing localization patterns and effects on gene expression [45]. Conversely, in seedlings the two hormones have synergistic effects, where interactions between the BR signaling repressor BIN2 and the auxin signaling repressor ARF2 can alter the seedling’s sensitivity to either hormone [46]. Additionally, high auxin levels from dominant overexpression *yucca* mutants can reduce the effects of BR on hypocotyl elongation by saturating growth responses [44]. In fact, our live imaging indicates that sepal elongation *drmy1* is more sensitive to BL or BRZ treatments than WT, which has much smaller changes in growth rates and patterns (Fig. 7). Perhaps the reduced auxin signaling levels in *drmy1* [18], while not altered by BR treatments (Additional file 1: Fig. S7c), render *drmy1* more susceptible to both endogenous and exogenous alterations to BR signaling. Additionally, perhaps BL does not alter outer sepal length because *drmy1* outer sepals have higher and more robust auxin levels [18] and elongation is already saturated while inner sepal elongation is not. It stands to reason, then, that inhibiting BR biosynthesis with BRZ would have a much larger impact on the outer sepal because it has more signaling to lose than the inner sepal.

It is also documented that cytokinin signaling is increased in *drmy1* because reduced translation levels lead to low protein levels of cytokinin signaling inhibitors including ARR7 and AHP6, which normally undergo rapid protein synthesis and degradation during sepal initiation [19]. It is possible that this same mechanism impacts inhibitors of BR signaling such as BIN2 to alter BR signaling levels and sensitivities. However, this regulation occurs at the translational level for these cytokinin pathway proteins and not at the transcriptional level, so such changes cannot be detected in our RNA-seq datasets and will require future investigation. Recent advances in BR research also suggests that uneven inheritance of BR levels in *Arabidopsis* root cell divisions can increase overall BR activity by circumventing the negative feedback loops typically associated with high levels of BR signaling [47]. It is possible that the higher variation in BR signaling (Fig. 5D) associated with *drmy1* creates a similar mechanism for spatially segregating BR biosynthesis from cells with high BR signaling, thus increasing BR sensitivity. In fact, our spatial RNA-seq dataset suggests that the BR biosynthesis gene *DWF1* is expressed most highly not in the regions with initiating sepals themselves, but in the adjacent spots just interior (Fig. 4G).

Although the untargeted spatial RNA-seq technology available when these experiments were performed did not have enough resolution to identify transcriptomic differences between different sepals (i.e. inner vs. outer vs. lateral sepals) on the same bud, growth rate and BES1 reporter experiments were able to verify broad trends in BR signaling levels and resolve the differences between different sepals. Future experiments could further address this by using newer untargeted methods with higher resolution such as Visium HD, slide-seq, stereo-seq, etc. or targeted methods such as Xenium and MERFISH [48–52]. Additionally, many published BR biosynthesis and signaling reporters (such as reporters for DWF4, CPD, BIN2, and BZR1) have little or no expression in flower buds, suggesting the promoters/enhancers guiding the expression patterns are more complex in these tissues. Future experiments will be crucial for identifying the link between *drmy1* and the increase in BR signaling noted here, as well as to understand the mechanism behind the differential responses between the outer and inner sepal to BR signaling levels.

## Conclusions

Results from this study reinforce the importance of coordinated growth patterns for proper organ development. It is clear the proper regulation of multiple hormones such as auxin, cytokinin, and brassinosteroids is crucial for robust growth. These results reveal a role for DRMY1-mediated BR signaling in coordinating inter-organ elongation, which is a largely unexplored area of plant development. This work paves the way for future studies to further investigate the complex dynamics and cross talk between hormones and how they lead to coordinated growth during sepal development.

## Methods

### Plant material

All genotypes are in the Columbia-0 background unless stated otherwise. The following lines were generated as previously described: *ap1 cal 35S::AP1-GR* (Ler background) [25, 53], *drmy1 ap1 cal 35S::AP1-GR* (Ler background, *drmy1* introgressed into Ler) [19], *35S::mCitrine-RCI2A* (pLH13) [54], *pUBQ10::mCherry-RCI2A* [18], *DR5::3xVENUS-N7* [55], *pUBQ10::BES1-ypet*/*pUBQ10::H2B-mCherry* [33]. *bzr1-1D* was previously described by Wang et al. [56]. *det2-1* (CS6159) was obtained from the Arabidopsis Biological Resource Center (ABRC).

### Plant growth

All *Arabidopsis* (*Arabidopsis thaliana*) were grown on Lambert Mix LM-111 soil. Seeds were sown on soil and stratified for about three days at 4 °C in darkness before transferring to the growth chambers. *ap1 cal 35S::AP1-GR* and *drmy1 ap1 cal 35S::AP1-GR* plants were grown in Percival growth chambers at 16 °C with constant illumination provided by Philips 800 Series 32 Watt fluorescent bulbs (f32t8/tl841) (∼100 µmol m^−2^ s^−1^). All other genotypes were grown in Percival growth chambers at 22 °C with 60% humidity under long day conditions (16 h light 8 h dark) provided by Philips 800 Series 32 Watt fluorescent bulbs (f32t8/tl841) (∼100 µmol m^−2^ s^−1^).

### Tissue collection

Once *ap1 cal 35S::AP1-GR *and *drmy1 ap1 cal 35S::AP1-GR* plants began to bolt, they were induced with 10 μM Dexamethasone in 0.015% Silwet (vol/vol) three times at roughly 24 h intervals. About 108 h (4 ½ days) after the first induction, when flower buds had reached stages 2–3, whole cauliflower-like inflorescences were cut and incubated in protoplasting solution, filtered, pelleted, and washed as described by Satterlee et al. [24] with the following adjustments: cells were incubated in protoplasting solution for 90 min, were passed through a 70 micron filter once and a 40 micron filter twice, and 5 μg/mL final concentration of fluorescein diacetate (FDA) was added after the final resuspension in 50–100 µL wash solution to stain live cells for counting. Two biological replicates were protoplasted per genotype using 0.25–0.6 g of tissue per replicate.

FDA stained cells were counted on a Countess 3 FL Automated Cell Counter (Invitrogen), diluted, and loaded onto the 10 × Genomics Chromium Controller using an 8 reaction 3’ HT kit v3 following the manufacturer’s instructions to target ∼10,000 cells per replicate. We believe cell counts were underestimated, as we obtained 20–25,000 cells per replicate despite targeting to load only 10,000.

For spatial RNA-seq with 10 × Visium, plants were induced the same way as for scRNA-seq. After 4 ½ days, small pieces of the floral meristem cluster were carefully removed and submerged in Tissue Tek® O.C.T. Compound (Sakura®) in a 10 × 10 × 5 mm cryomold. Air bubbles were carefully removed with a 200 µL pipette tip and 9–13 cluster pieces from separate plants serving as biological replicates were arranged on the bottom of the cryomold. O.C.T. embedded tissue was then flash frozen in liquid nitrogen cooled isopentane (2-methylbutane) and stored at −70 °C until cryosectioning.

### Single cell RNA-seq processing

Single cell libraries were sequenced on two P3 flow cells on Illumina NextSeq 2 k 100 bp kits. Raw single cell data reads were converted to fastq format with cellranger v7.0.1 mkfastq and converted to feature matrices with cellranger count using default settings. We used a custom reference by running the TAIR10 v6.0.2 Arabidopsis genome through cellranger mkref using default settings. Matrices were loaded into R version 4.3.0 and analyzed in Seurat v4 [57]. Data was filtered to only include cells expressing greater than 600 but less than 6000 genes (Additional file 1: Fig. S11a) and with less than 10% mitochondrial transcripts and 20% chloroplast transcripts. For the remaining cells, PercentageFeatureSet was used to calculate the percentage of transcripts originating from mitochondrial genes (Additional file 1: Fig. S11b) and chloroplast genes (Additional file 1: Fig. S2b) for later visualization. Cell cycle was scored using a list of cell cycle genes from Zhang et al. [58] (Additional file 5: Table S9) and the CellCycleScoring function in Seurat. To remove the effects of cell cycle on clustering (Additional file 1: Fig. S2a), the percent variance explained by cell cycle phase for each gene was calculated using the GetVarianceExplained function from the R library “scater” and any gene with > 3% variance explained by cell cycle (1,189 genes total) was deleted from the dataset (Additional file 2: Table S1). Mitochondrial genes were also removed. To check the effects of protoplasting, we used the bulk RNA-seq of WT and *drmy1* in the *ap1cal 35S::AP1-GR* background published in Kong et al. [19] to check for genes expressed in our single cell dataset that are not expressed in the bulk RNA-seq dataset. We identified 2,421 genes in WT and 2,522 genes in *drmy1* that were potentially induced by protoplasting with this method.

The top 5000 variable features were selected with FindVariableFeatures set to the selection method “vst”. Data was transformed with SCTransform version 2 with 5000 variable features. WT and *drmy1* samples were integrated using FindIntegrationAnchors and with normalization.method set to “SCT” and anchor.features chosen with the command SelectIntegrationFeatures. The anchors were then used with the command IntegrateData set to dims = 1:50 and normalization.method set to “SCT”.

### Dimensionality reduction

UMAPS were generated in seurat from the integrated scRNA-seq data by scaling the data with ScaleData followed by RunPCA set to npcs = 50 and RunUMAP on all 50 principal components. Clustering was performed with FindNeighbors (dims = 1:50) and FindClusters (resolution = 0.4).

### Cluster annotation

Clusters were annotated by viewing marker gene expression patterns with the built in Seurat functions FeaturePlot and VlnPlot. Marker genes were chosen from several published single cell references [30, 58, 59] (Additional file 2: Table S2) and additional marker genes were identified using the “scoreMarkers” function from the scoreMarkers package and setting the mean Area Under the Curve (AUC) threshold to 0.7. (Additional file 5: Table S10). The most informative markers were then visualized with the dotplot function in Seurat using SCT normalized expression values. Conserved markers for cluster 2 (boundaries) were identified by preparing the scRNA-seq Seurat object with the “prepSCTFindMarkers” function in Seurat followed by the “FindConservedMarkers” function.

Floral meristem atlas mapping was performed as described in Neumann et al. [30]. Briefly, the pipeline uses novosparc to map scRNA-seq cells onto physical cells from a floral meristem atlas by computing cost matrices for gene expression “distances”, location physical distances, and computing optimal transport of the cells onto locations based on the cost matrices (see [30, 60] for more details). The probability score is based on these cost matrices. For each cell in the floral meristem atlas, these distances are normalized such that the sum of the distances to all of the potential single cell cluster identities are 1. So hypothetically the range is 0 to 1, although in reality it is more relevant/important to consider the value of the probability score for a given atlas cell in relation to the value of the other atlas cells as opposed to the entire hypothetical range. E.g. the cells with the highest relative probabilities compared to other cells likely represent that single cell cluster, while cells with probabilities at the lower end of the relative scale are likely not related to that same cluster. For our dataset, we set “pre_mode” to “expr_pre” (to enrich for cells that map best to the atlas) and set other parameters as follows: ns = 10, nt = 4, ap = 0.1, ep = 0.05, tr = “none”, md = “euclidian”, ts = 100,000, tc = 100, mi = 5000, to = 1e-9, ms = 0, and ce = False. The rest of the pipeline was kept the same as the provided scripts.

Single cell metadata including individual cell barcodes, seurat cluster annotation, cell cycle scoring and assignment, mitochondrial and chloroplast reads, etc. are available in Additional file 6: Table S11.

### Gene expression analysis

Lists of genes that are up and down-regulated in Arabidopsis seedlings in response to exogenously applied hormones were downloaded from Nemhauser et al. [31]. PercentageFeatureSet was used to calculate the percentage of transcripts in each cell that originate from each list of up or down-regulated genes and plotted with FeaturePlot, VlnPlot, and Dotplot. For dotplots, the dot size was determined by the log base 10 of the inverse of the p-value, with a bonferroni corrected significance threshold of p < 0.000245. Dots with p-values above this threshold were not displayed. Dot color was determined by subtracting the mean value for WT samples from the mean value for *drmy1* samples—thus higher positive numbers represent higher expression levels in WT and higher negative values represent higher expression levels in *drmy1*.

### GO term enrichment and gene set enrichment analysis

We pseudobulked the scRNA-seq dataset using the AggregateExpression function in Seurat, grouped by cluster identity and by replicate. Then differential gene expression was performed using the DEseq function in DEseq2 with default parameters (Additional file 3: Table S3). Since it is unknown whether DRMY1 can regulate gene expression directly through DNA binding, we do not speculate on which differentially expressed genes may be direct or indirect regulatory targets in downstream analyses. To perform Gene Ontology (GO) Term enrichment on differentially expressed (DE) genes, the DE gene list was first filtered to genes with adjusted p-values less than 0.05, then we imported GO Terms from TAIR “ATH_GO_GOSLIM.txt” into R. We performed GO Term Enrichment for each pseudobulked single cell cluster with the GSEA function (Gene Set Enrichment Analysis) from the clusterProfiler bioconductor package [61], setting the maxGSSize to 5000 and the pvalueCutoff to 0.05. The background gene set was the entire set of annotated genes in Arabidopsis (as annotated in ATH_GO_GOSLIM.txt). The results were visualized with a dotplot in ggplot2, with GO terms and clusters arranged according to similarity using hierarchical clustering via the hclust function in R. A dendrogram for the GO Terms was created using the ggtree function from the ggtree library [62]. GSEA was performed in a similar manner for determining whether hormone up and down-regulated genes are enriched in *drmy1*. The same gene lists from Nemhauser et al. [31] for hormone up and down-regulated genes were used in place of ATH_GO_GOSLIM.txt file. In addition, any gene with one of the hormones mentioned in any of its GO terms was pulled into a list and annotated as “[hormone]_GO” to create a list of genes associated with that hormone. Results were visualized with dotplots.

### Cryosectioning for Spatial RNA-seq

Fresh frozen floral meristem clusters stored at −70 °C were equilibrated to −10 °C in a Thermofischer HM 525 NX cryostat for about 30 min. Tissue blocks were cut into 10 micron thick sections and placed on each capture area according to the instructions in the 10 × Visium User Guide. Each capture area had a section from a separate block of frozen tissue. For optimization slides, we put 4 *drmy1 ap1 cal 35S::AP1-GR* sections and 4 WT *ap1 cal 35S::AP1-GR* sections on each slide, while the spatial gene expression slide had 2 sections from each genotype, with each section containing 9 to 13 cauliflower-like clusters to serve as biological replicates.

### Optimization for spatial RNA-seq

To optimize RNA permeabilization, we used the Visium Spatial Tissue Optimization Slide & Reagent Kit, 4 slides (10 × Genomics PN-1000193). We followed the optimization protocol included with the kit with the following adjustments. For all four optimization slides, we skipped the H&E staining steps, instead either not staining tissue (Slide 1) or staining in 0.5 mg/mL toluidine blue in 3.5% ethanol 96.5% water for 30 s—4 min. Slides were briefly dipped in 1 × PBS buffer prepared with RNAse free water to rinse off excess toluidine blue, then extra liquid was allowed to drip off the slide. For slides 2, 3, and 4 we added a pre-permeabilization step according to the instructions in [63]. Briefly, immediately prior to permeabilization we incubate the capture areas in 70 uL of pre-permeabilization mixture (2% Polyvinylpyrrolidone-40, 1 × Exonuclease I buffer, 0.19 ug/uL BSA) at 37 C for 30 min, then rinsed in 100 uL 0.1 × SSC buffer. Various permeabilization times between 1 and 30 min were tested, with 4–5 min determined to be optimal (Additional file 1: Fig. S12). Brightfield imaging was performed either on a Microbeam laser capture microdissection system (Zeiss) with the 20 × objective or a Keyence BZ-X810 with the 20 × objective (0.45 NA). Fluorescent cDNA was imaged either on the Keyence BZ-X810 with TRITC filter under the 20 × objective (0.45 NA) or on a Zeiss 710 upright confocal microscope with a 20 × Plan-Apochromat water-dipping lens (1.0 NA).

### Spatial RNA-seq

Visium spatial RNA-seq was performed according to the instructions in the user manual with the following notes and alterations. One cryosection slice was placed on each capture area of the Visium slide, each containing approximately 9–13 WT *ap1 cal 35S::AP1-GR* or *drmy1 ap1 cal 35S::AP1-GR* cauliflower-like clusters. After methanol fixation, H&E staining was replaced with toluidine blue staining as described above, then a coverslip was mounted with a solution of 85% glycerol, 10% RNAse free water, and 0.5% RNAseOUT, followed immediately by brightfield imaging as described above. Pre-permeabilization was performed as described above, and then the user manual was followed for the rest of the protocol using a 5 min permeabilization time.

### Spatial RNA-seq processing

Paired end libraries were sequenced on one P2 flow cell on an Illumina NextSeq 2 k 100 bp kit and one mid-output flowcell on an Illumina NextSeq500 150 bp kit. Reads were converted to fastq format with spaceranger mkfastq and analyzed with spaceranger count v2.0 with default settings. We used a custom reference by running the TAIR10 v6.0.2 Arabidopsis genome through cellranger v7.0.1 mkref using default settings. Brightfield images of capture areas were manually aligned to the capture spots and fiduciary frame in cLoupe browser v6.4.1.

### Spatial deconvolution

Deconvolution was performed in Seurat v4 by first merging data from all four capture areas, then normalizing with SCTransform (assay = “Spatial”) and creating a PCA reduction with RunPCA. FindTransferAnchors was used to find anchors between the single cell dataset and the Visium dataset, then a predictions assay was generated with the TransferData function using the anchors, single cell cluster identities as the refdata, and the Visium PCA reduction as the weight.reduction, and dims = 1:30. For quality control, we visualized the number of features, UMIs, and percent mitochondrial transcripts on the samples. We found that one of the capture areas with a WT section had abnormally higher mitochondrial transcripts than the others (Additional file 1: Fig. S5) suggesting cells in this section may have been dying, so we omitted this capture area from downstream analyses.

We used SpatialFeaturePlot to visualize the predicted mapping of each single cell cluster onto the Visium spots. For each Visium spot, we identified which single cell cluster had the highest prediction score and entered this cluster as a new metadata feature and visualized it with SpatialDimPlot. The percentage of transcripts from various hormone up and downregulated genes was calculated for each Visium spot using the same process as for the single cell dataset. The results were visualized using SpatialFeaturePlot, and in order to generate plots with the same scale for each capture area, we added “& scale_fill_viridis_c(option = "turbo", limits = c(0,upper_limit), oob = scales::squish))” where “upper_limit” is the maximum percentage found among all Visium spots.

To verify the ability of the prediction scores to identify boundary identity, we also generated module scores by sorting the single cell markers for cluster 2 (boundaries) identified with FindConservedMarkers based on “max_pval”. We took a list of the 100 genes with the lowest max p-value (Additional file 5: Table S7) and used that list as the features in the AddModuleScore function in Seurat, which created a module score with that gene set for each Visium spot.

### Brassinazole and brassinolide experiments

WT and *drmy1* (Col-0) plants expressing the pLH13 (*35S::mCitrine-RCI2A*) membrane marker were used right when they began to bolt (bolts 0–3 cm in height). Hormone solutions contained 0.0025% (v/v) Silwet and either 50 μM brassinazole (50 mM stocks dissolved in DMSO or DMF) (VWR TCB2829-100MG), 400 nM brassinolide (400 μM stock dissolved in DMSO or DMF) (Cayman Chemicals CAYM-21594–25), or 0.1% DMSO or DMF (mock treatments). Plants were dipped in 100 mL baths of their respective treatments’ solution once daily for 1 min for 6 days. On the 7th day, inflorescences were dissected under a Zeiss Stemi 2000-C Stereo Microscope with a Dumont tweezer (Electron Microscopy Sciences, style 5, no. 72701-D) down to stage 6 or earlier flower buds [29]. Dissected inflorescences were placed on 60 mm × 15 mm petri dishes (VWR 25384–092) with live imaging media modified from [64]. The media was made with 0.5 × MS basal salts, 1 × Gamborg vitamin mixture, 1% w/v sucrose, 1 mg/mL MES buffer, 1.2% w/v phytoagar (Fisher), and 0.1% Plant Preservative Mixture (Plant Cell Technology) and pH adjusted with 1 M KOH to 5.8. For silique images from Additional file 1: Fig. S7a, 11 days after the first BRZ/BL treatment, the earliest stage 17 siliques and their pedicels were removed from the stem and imaged under a Zeiss Stemi 508 stereomicroscope using a Schott KL 300 LED light source and an Accu-scope camera with CaptaVision + software.

For single timepoint experiments, plants were imaged immediately on a Zeiss 710 upright confocal microscope with a 20 × Plan-Apochromat water-dipping lens (1.0 NA). Due to frequent silencing of the pLH13 membrane marker, plants in single timepoint experiments were stained with a 0.1 mg/mL propidium iodide and 0.1% Silwet solution for 5 min and rinsed 3–5 times in DI water before imaging.

For live imaging experiments, the media plates also contained either 5 μM brassinazole, 40 nM brassinolide, or 0.1% v/v DMF (mock). Images were taken on a Leica Stellaris 5 confocal microscope with a 25 × Plan-Apochromat water-dipping lens (0.95 NA) every 12 h for 36 to 60 h. Between timepoints, samples were placed back in the same long day growth chamber plants were originally grown in (see “Plant Growth”).

The following laser and wavelength were used in confocal imaging. For chlorophyll, excitation 488 nm, emission 647–721 nm. For PI, excitation 514 nm, emission 566–650 nm. mCherry, excitation 594 nm, emission 600–659 nm. For mCitrine/ypet/VENUS, in *35S::mCitrine-RCI2A*, excitation 514 nm, emission 519–580 nm; in *DR5::3xVENUS-N7* and *pCUC1::3xVenus-N7*, excitation 514 nm, emission 519–569 nm.

### Cycloheximide treatment

Cycloheximide treatments were performed as described in Kong et al. [19].

### Image analysis

For single timepoint measurements of sepal lengths and outer/inner sepal ratios, confocal image stacks were first converted to maximum intensity projections. These projections were loaded into Fiji v1.53c [65] and the scale was set using the scale bar included in the projections. The line tool was used to measure the length from the back (proximal side) of the sepal to the longest point of the sepal tip (distal side) for inner and outer sepals. The diameter (length) of the entire bud from the back of the outer sepal to the back of the inner sepal was also measured (Fig. 6B). Boxplots were generated in R with ggplot2 and statistics calculated with a Wilcoxon two sided signed rank test.

For live imaging experiments, early stage 3 buds that had just initiated outer sepals were chosen to serve as the 0 h timepoint for quantifying sepal growth. To help control for sepal initiation timing variability in *drmy1*, buds that had not begun initiating inner sepals at the 12 h timepoint were excluded from analysis. Confocal stacks were once loaded into ImageJ and converted to .tif files before loading into MorphoGraphX 2 [66, 67]. Images were brightened once or twice with Stack/Filter/Brighten Darken (amount = 2) and blurred with Stack/Filters/Gaussian Blur Stack (x = y = z = 1) 2–3 times. Stack/Lyon/Init level set (up threshold = down threshold = 2) was run followed by Stack/Lyon/Level Set Evolve (alpha = 0, beta = 0.1, lamda = 10, dt = 100, epsilon = 1.5, threshold = 0.002, max steps = 5, view steps = 5) 2–3 times. Stack/Morphology/Edge Detect Angle (fill holes = no) was then applied, and Stack/Morphology/Closing (x = y = z = 3, Round = no) used to close holes. Any material outside of the bud of interest was manually removed using the voxel edit tool. A mesh was created using Mesh/Creation/Marching Cubes Surface (cube size = 5, threshold = 20,000), then refined using Mesh/Structure/Smooth Mesh (passes = 10, walls only = no) and Mesh/Structure/Subdivide functions three times each. Cell outlines were projected onto the mesh using Mesh/Signal/Project Signal (use absolute = no, min signal = 0, max signal = 60,000) with a variable depth optimized to capture the best signal from epidermal cells from each bud—most often min distance = 1 μm and max distance = 3 μm was ideal. For segmentations of every cell, Mesh/Signal/Gaussian Blur (radius = 0.5 μm) was used to blur the cell membrane signal slightly, then cells were manually seeded, then segmented with Mesh/Segmentation/Watershed Segmentation (steps = 50,000) and Mesh/Segmentation/Fix Corner Triangles. For analyses where entire sepal growth rates were analyzed, each sepal was segmented as if it were one big cell. This was achieved by manually defining the boundaries of the sepals at the latest (36 h) timepoints following existing cell boundaries. Then earlier timepoints were segmented by following the same cell boundaries to create the sepal boundaries.

To calculate growth rates between timepoints, a previous timepoint would be loaded as a second mesh and parent labels manually set by aligning the two buds and using the “grab labels from other surface” tool. Errors in parent tracking and segmentation were identified using Mesh/Cell Axis/PDG/Check Correspondence and manually fixed by deleting labels and re-segmenting as needed. Heat maps were made using Heat Map Classic with type set to Area, visualization to geometry, “change map” option checked with “ratio” and “decreasing” chosen in the drop down menus and “use manual range” set to minimum 0 maximum 2.5. For whole sepal analyses, the growth rate heatmaps were exported using Mesh/Attributes/Save to CSV extended. Absolute growth amounts were calculated by copying the area of each sepal from the previous timepoint into the CSV file and subtracting the current timepoint’s area from the previous area for each sepal. Percentage contributions to total sepal growth were calculated by summing the absolute growth amounts from all sepals, then dividing each sepal’s absolute growth by the summed growth. Sepal ratios were calculated by simply dividing the area of the outer sepal by the area of each other individual sepal. However, in cases where there were two inner sepals instead of one, both inner sepals together were treated as one sepal to prevent inflating the outer/inner sepal ratio.

For BES1/H2B ratiometric images, confocal stacks were once again converted to .tif files to load into MorphoGraphX. The H2B channel was loaded first, and the main stack was copied to the work stack and signal outside of the bud(s) of interest was removed with the voxel edit tool. Stack/Filters/Gaussian Blur Stack (x = y = z radius = 0.8 μm) was applied, then seeds created with Stack/Segmentation/Detect Local Maxima (x = y = z radius = 0.5, start label = 2, threshold = 3000, value = 60,000). Meshes were created with Mesh/Creation/Mesh From Local Maxima (radius = 2 μm). Many overlapping nuclei meshes are inadvertently created with this method, in which case one overlapping nucleus is chosen at random and manually removed by removing the label, using Mesh/Selection/Select Unlabeled, and hitting backspace to delete. Labels were added to each bud corresponding to their developmental stage by using the lasso tool to select all of the nuclei in a bud and using Mesh/Lineage Tracking/Set Parent to assign a unique numeric parent label based on the bud’s stage. Mesh/Signal/Project Signal (min = 0 μm, max = 2 μm, min signal = 0, max signal = 100,000, use absolute = no) projected the nuclear H2B signal onto the meshes, which was then quantified with Mesh/Heat map/Measures/Signal/Signal Total. Then the BES1 channel was loaded into MorphoGraphX and projected onto the mesh in the same way, but quantified using Mesh/Heat map/Measures/Signal/Signal Interior. This quantification is functionally the same as Signal Total in this case, but allows us to store the BES1 signal as a separate attribute, at which point both can be exported together with Mesh/Attributes/Save to CSV extended. The BES1/H2B signal ratio was calculated for each ratio by dividing the Signal Interior column (BES1) by the Signal Total column (H2B). The ratio can be visualized by using Mesh/Heat map/Heat Map Load and choosing the ratio column, and using Mesh/Heat map/Heat Map Set Range (min = 0 max = 4) to make the scale consistent between images.

Sepal initiation timing was performed as detailed in [19].

## Supplementary Information

Additional file 1. Supplementary figures S1 to S12 and corresponding legends.

Additional file 2: Table S1. List of genes with >3% variance explained by cell cycle phase and removed from single cell RNAseq dataset. Table S2: Marker genes used to help identify single cell RNAseq clusters.

Additional file 3: Table S3. Output of DEseq2 differential gene expression analysis on pseudobulked scRNA-seq dataset.

Additional file 4: Table S4. Output of Gene Set Enrichment Analysis for gene ontology term enrichment.

Additional file 5: Table S5. List of genes from Nemhauser et al. up and downregulated by application of various hormones. Table S6. Statistical information for dotplots in Fig. S3a-c, Fig. 3b,c, and Fig. 4h. Table S7. Output of Seurat FindConservedMarkers to identify markers for cluster 2. Table S8. ANOVA results and Tukey's Honest Significant Difference test results from Fig. 5. Table S9. A list of cell cycle marker genes used to annotate the cell cycle phases. Table S10. Output of ScoreMarkers used to identify additional single cell cluster specific marker genes.

Additional file 6: Table S11. Metadata from single cell-RNAseq.

## Data Availability

The datasets supporting the conclusions of this article are available in the National Center for Biotechnology Information Gene Expression Omnibus repository under the accession number GSE320437 [68] (For spatial RNA-seq Datasets) and GSE303396 [69] (For single cell RNA-seq). Scripts for analyzing data can be accessed via GitHub [70] at https://github.com/RoederLab/drmy1_BR.git or Zenodo [71] at https://doi.org/10.5281/zenodo.20510622 under a MIT license. Confocal microscopy images from this manuscript can also be obtained at the same Zenodo repository [71]. The following Arabidopsis lines are available through the Arabidopsis Biological Resource Center (ABRC) under stock numbers: *35S::mCitrine-RCI2A* (CS74153), *drmy1* (CS74140), *drmy1 35S::mCitrine-RCI2A* (CS74142), *drmy1 ap1 cal 35S::AP1-GR* (CS74143), *det2 35S::mCitrine-RCI2A* (CS74138), *det2 drmy1 35S::mCitrine-RCI2A* (CS74137),* bzr1-1D drmy1 35S::mCitrine-RCI2A* (CS74139).
