# Supplementary material for: Spatial and single-cell transcriptomics uncover brassinosteroid-mediated coordination of sepal elongation in Arabidopsis
Source: Genome Biol. 2026 Jun 17;27:271. doi: 10.1186/s13059-026-04156-1 (PMC13508305; doi:10.1186/s13059-026-04156-1)
Supplement: Supplementary file 1 [file 13059_2026_4156_MOESM1_ESM.pdf]

Supplementary Figures

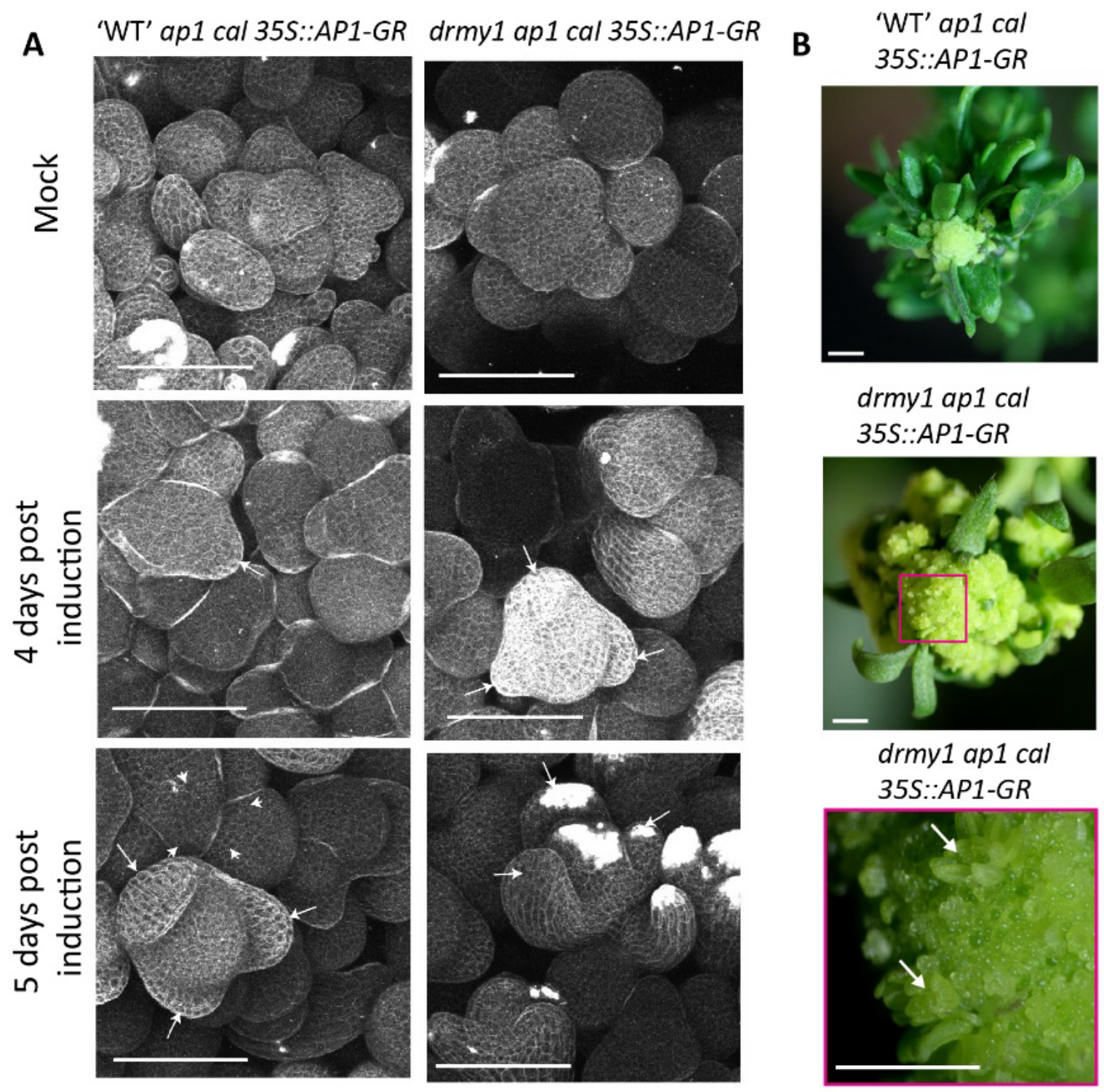

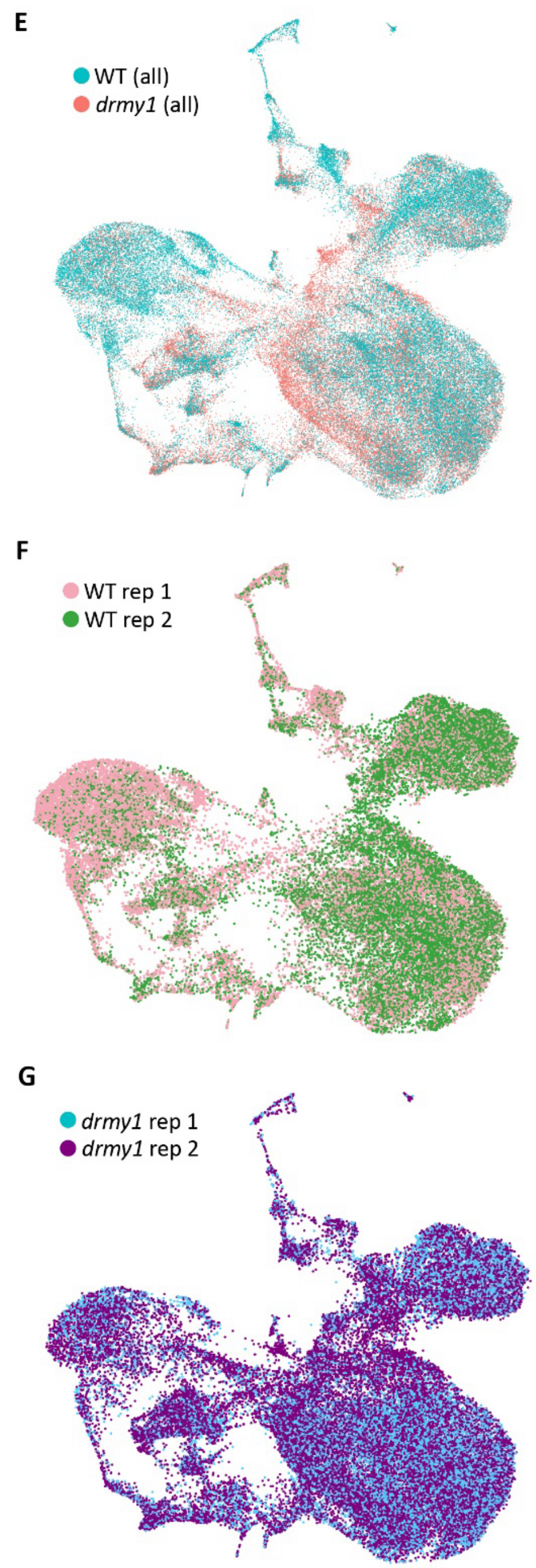

C

	# Cells	Mean UMI/cell	Mean genes/cell
WT	41,957	6,985	2,721
drmy1	51,408	6,027	2,525
Total	93,653		22,854
Avg		6,457	2,613

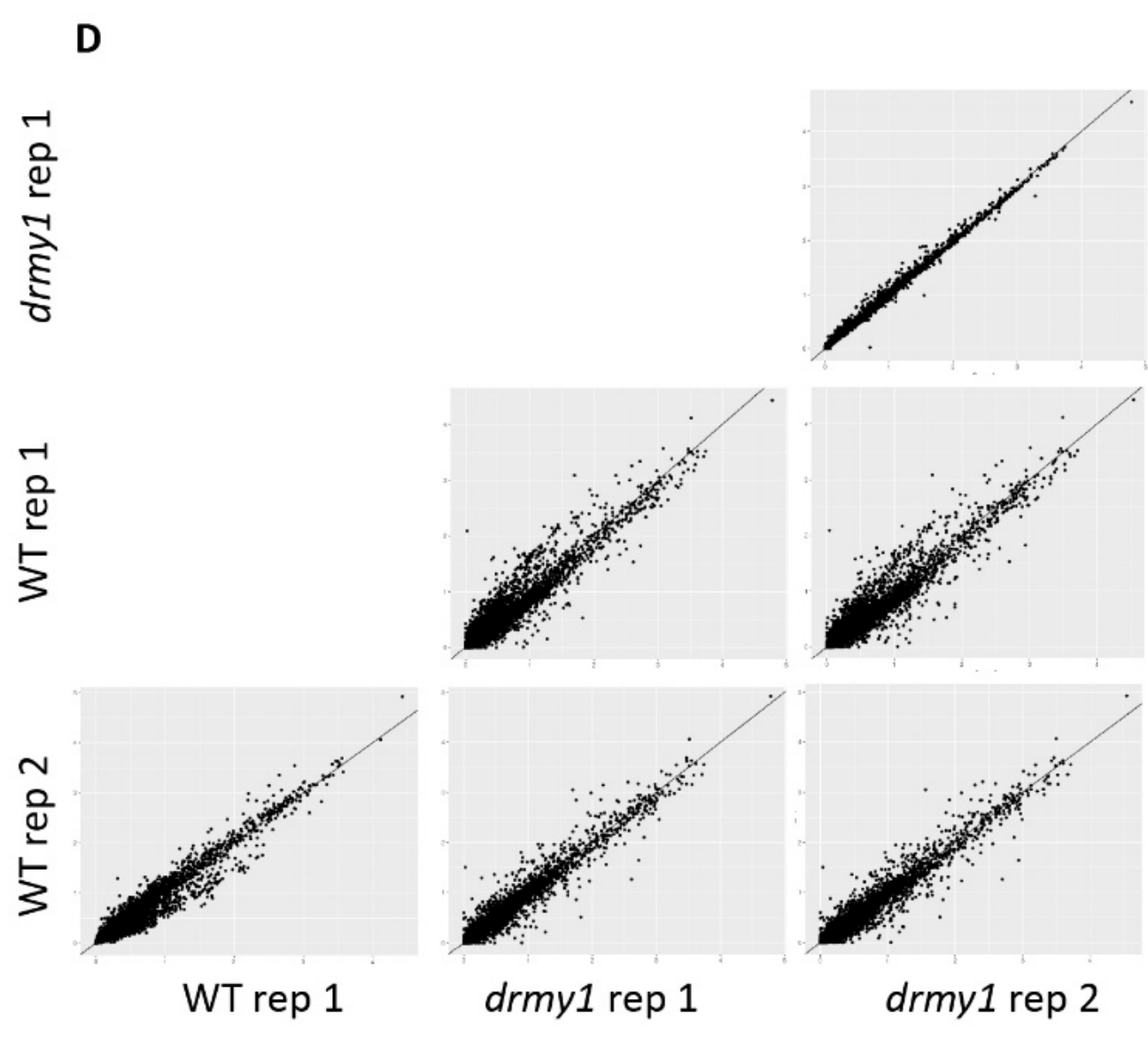

Fig. S1: Single cell RNAseq quality control

A) Confocal images of propidium iodide stained WT and *drmy1* floral meristem clusters in the *ap1 cal 35S::AP1-GR* background 4 days and 5 days after induction with 10 μM dexamethasone (dex) or mock. Examples of initiating sepals marked with arrows.

B) Stereomicroscope images of pre-induction WT and *drmy1* floral meristem clusters in the *ap1 cal 35S::AP1-GR* background. Note the larger numbers of leafy bracts present in WT, also note the older "escaped" flowers in *drmy1* that began initiation before dex induction marked by arrows.

C) Table of cell counts, mean unique molecular identifiers (UMIs), and mean number of genes detected per cell.

D) Correlation between the average normalized expression of each gene in WT and *drmy1* replicates.

E) UMAP projection color coded by genotype, with WT cells placed on top of *drmy1* cells.

F-G) UMAP projection of WT (F) or *drmy1* (G) cells color coded by replicate.

Scale bars 100 μm (A), 1 mm (B)

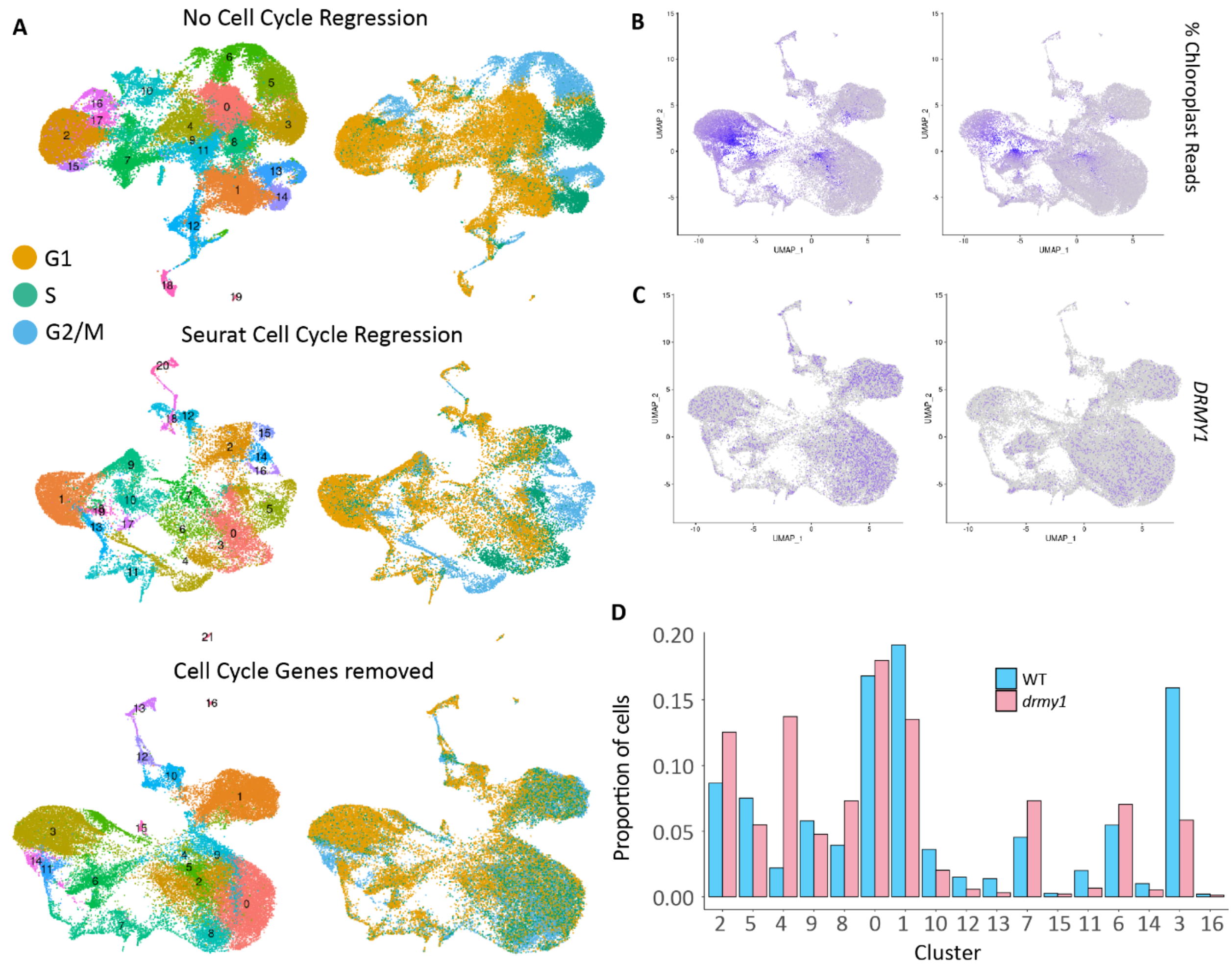

Fig. S2: Single cell RNAseq cell cycle regression, cell type composition, and gene expression.

A) UMAPs showing the clustering (left column) and cell cycle phase (right column) of each cell, demonstrating the effects of cell cycle on clustering and how different methods of removing cell cycle effects (no regression on top, Seurat cell cycle regression using the "vars.to.regress" argument in the ScaleData function in the middle, and complete removal of genes with > 3% variance explained by cell cycle phase on the bottom). The complete removal of cell cycle genes was used for further analysis.

B) Feature plots of the percentage of transcripts from chloroplast genes. Note the relatively high proportion of chloroplast genes in cluster 3, which helped identify this as likely mesophyll.

C) SCT normalized expression of *DRMY1* in each cell.

D) Proportion of cells from each cluster in WT and *drmy1* single cell data.

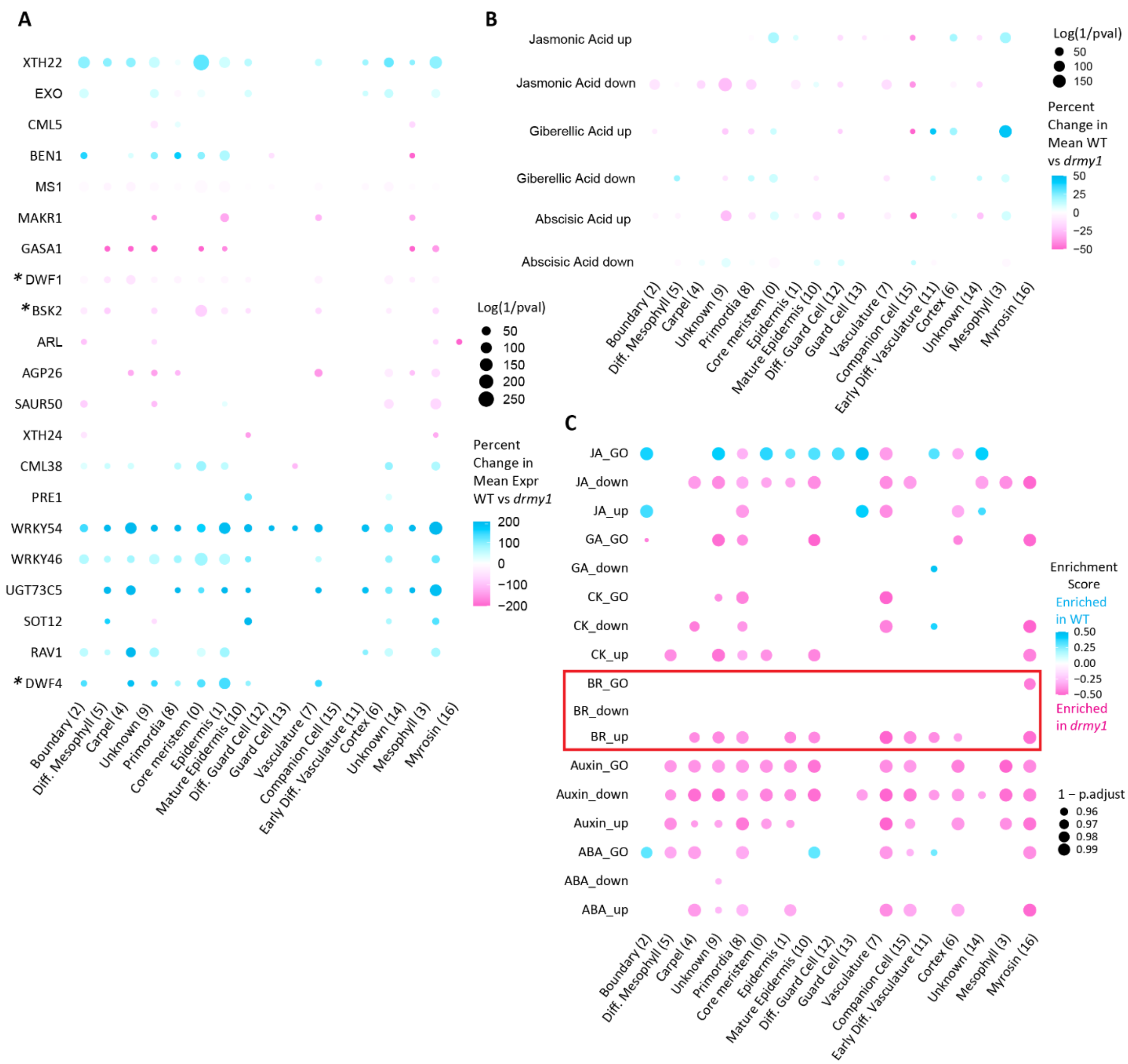

Fig. S3: Hormones up and downregulated in *drmy1*.

A) Dotplot showing the differences in expression of genes differentially expressed in *drmy1* with gene ontology terms that mention brassinosteroids. The size of each dot represents the log of the inverse of the p-value between the mean expression of the two genotypes with a Bonferroni adjusted threshold of $p < 0.00014$. See Additional file 5: Table S6 for exact p-values. The color of each dot represents the

percentage increase or decrease of the mean of *drmy1* compared to WT. Stars indicate genes in the brassinosteroid biosynthesis or signaling pathways, which includes *DWF1* (biosynthesis), *DWF4* (Biosynthesis, negatively regulated by brassinosteroids), and *BSK2* (signaling).

B) Dotplot showing the differences in expression of genes up and down-regulated by jasmonic acid, abscisic acid, and gibberellic acid. For each cell, the percentage of transcripts that come from the set of genes up or downregulated by applications of these hormones according to Nemhauser et al. (2006) is calculated, then the mean of all the cells in each cluster is calculated for WT and *drmy1*. The size of each dot represents the log of the inverse of the p-value between the mean of the two genotypes with a Bonferroni adjusted threshold of $p < 0.000245$. See Additional file 5: Table S6 for exact p-values. The color of each dot represents the percentage increase or decrease of the mean of *drmy1* compared to WT: blue represents higher expression levels in WT and pink represents higher expression levels in *drmy1*

C) Dotplot showing the results of Gene Set Enrichment Analysis (GSEA) on the sets of genes up or downregulated by applications of several hormones according to Nemhauser et al. (2006), as well as on the set of genes that contain the name of a given hormone in any of their GO terms ("hormone_GO" rows). The size of each dot represents 1 minus the adjusted p-value. Blue dots represent higher enrichment scores in WT and pink represents higher enrichment scores in *drmy1*. See Additional file 5: Table S6 for exact p-values and enrichment scores. Red box is used to highlight the brassinosteroid gene sets.

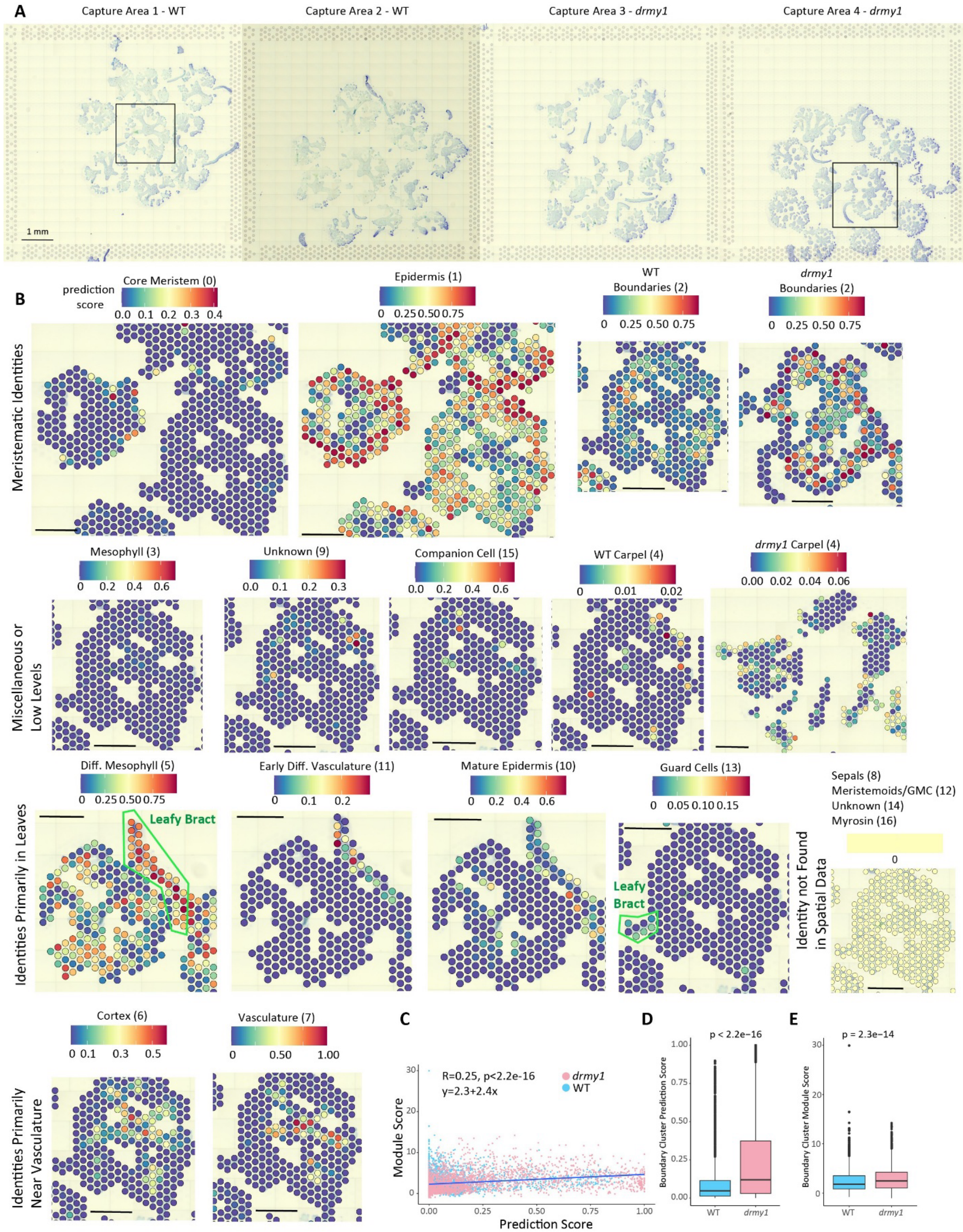

Fig. S4: Cell identity patterns in Visium spatial RNAseq from spatial deconvolution.

A) Brightfield images of all four Visium capture areas. Boxes show the representative areas used for visualizing trends in Fig. 3. Scale bar 1 mm.

B) Prediction scores of how well each single cell cluster maps to each Visium spot. Organized by similar expression patterns. All sections are wild type except as indicated for the boundary cluster (2) and developing carpel cluster (4). Scale bars 500 µm.

C) Scatterplot of prediction scores for boundary cluster identity vs module scores for boundary cluster identity for each Visium spot. n = 4081.

D) Boxplot of Seurat prediction scores for each Visium spot in WT and *drmy1*. n = 1421 (WT), n = 2660 (*drmy1*). $p<2.2 \times 10^{-16}$ in Wilcoxon's rank sum test.

E) Boxplot of Seurat module scores for each Visium spot in WT and *drmy1*. Module scores were generated using the top 100 conserved markers for cluster 2 (boundaries) in the single cell dataset (see methods). n = 1421 (WT), n = 2660 (*drmy1*). $p=2.3 \times 10^{-14}$ in Wilcoxon's rank sum test.

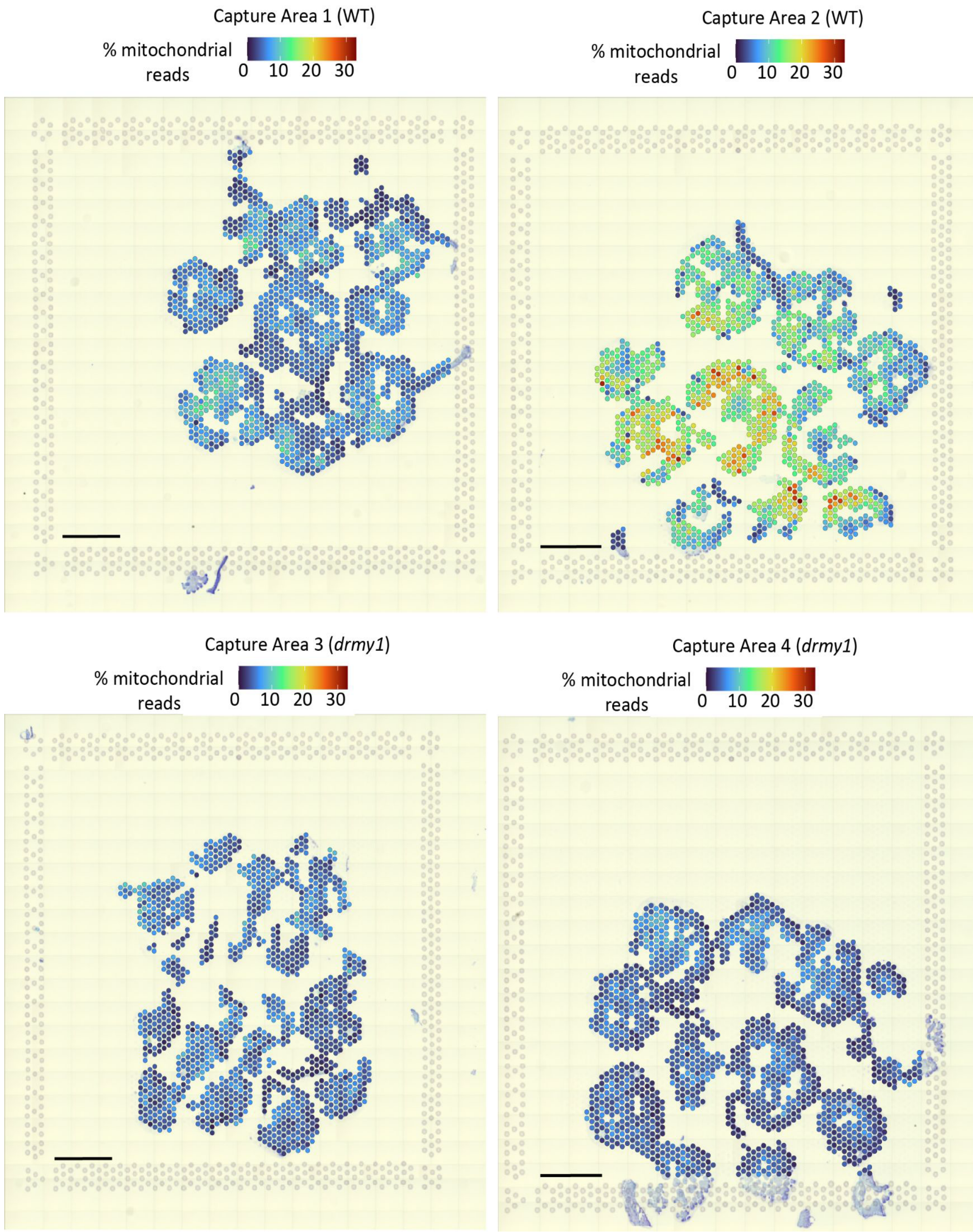

Fig. S5: WT capture area 2 has high numbers of mitochondrial transcripts

Percentage of transcripts coming from mitochondrial genes. Note how capture area 2 (a WT section) has much higher overall mitochondrial read percentages. This capture area was excluded from further analysis.

Scale bars 1 mm

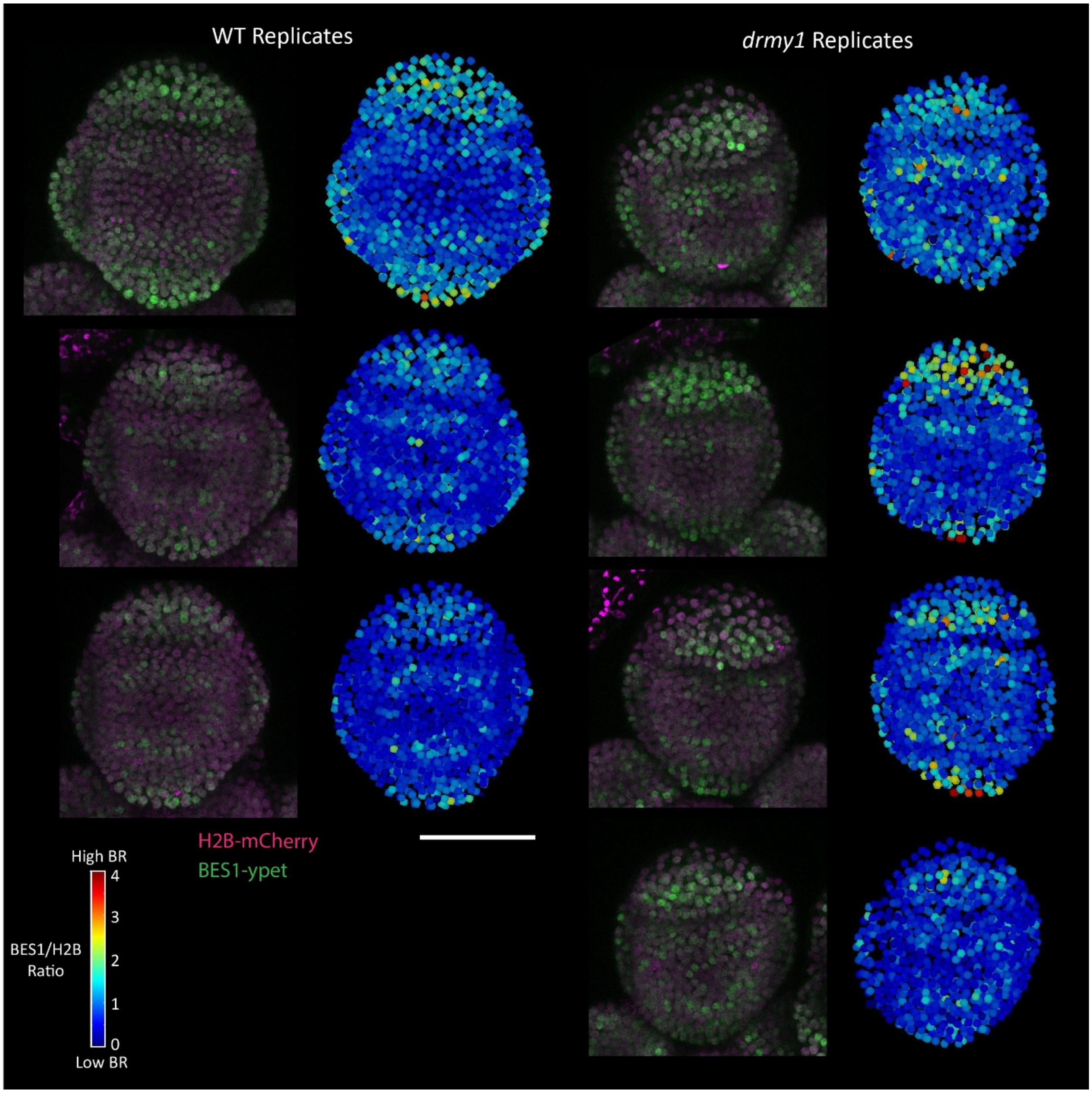

Fig. S6: *drmy1* has significant variability in BR signaling across replicates.

Additional replicates associated with Fig. 4a-d. Confocal maximum intensity projections of WT and *drmy1* expressing *pUBQ10::H2B-mcherry* (magenta)/*pUBQ10::BES1-ypet* (green) and the ratio of the

total BES1-ypet signal divided by the total H2B-mCherry signal in each nucleus of stage 3 buds. Note the variability in BES1/H2B ratios.

Scale bar 50 μm

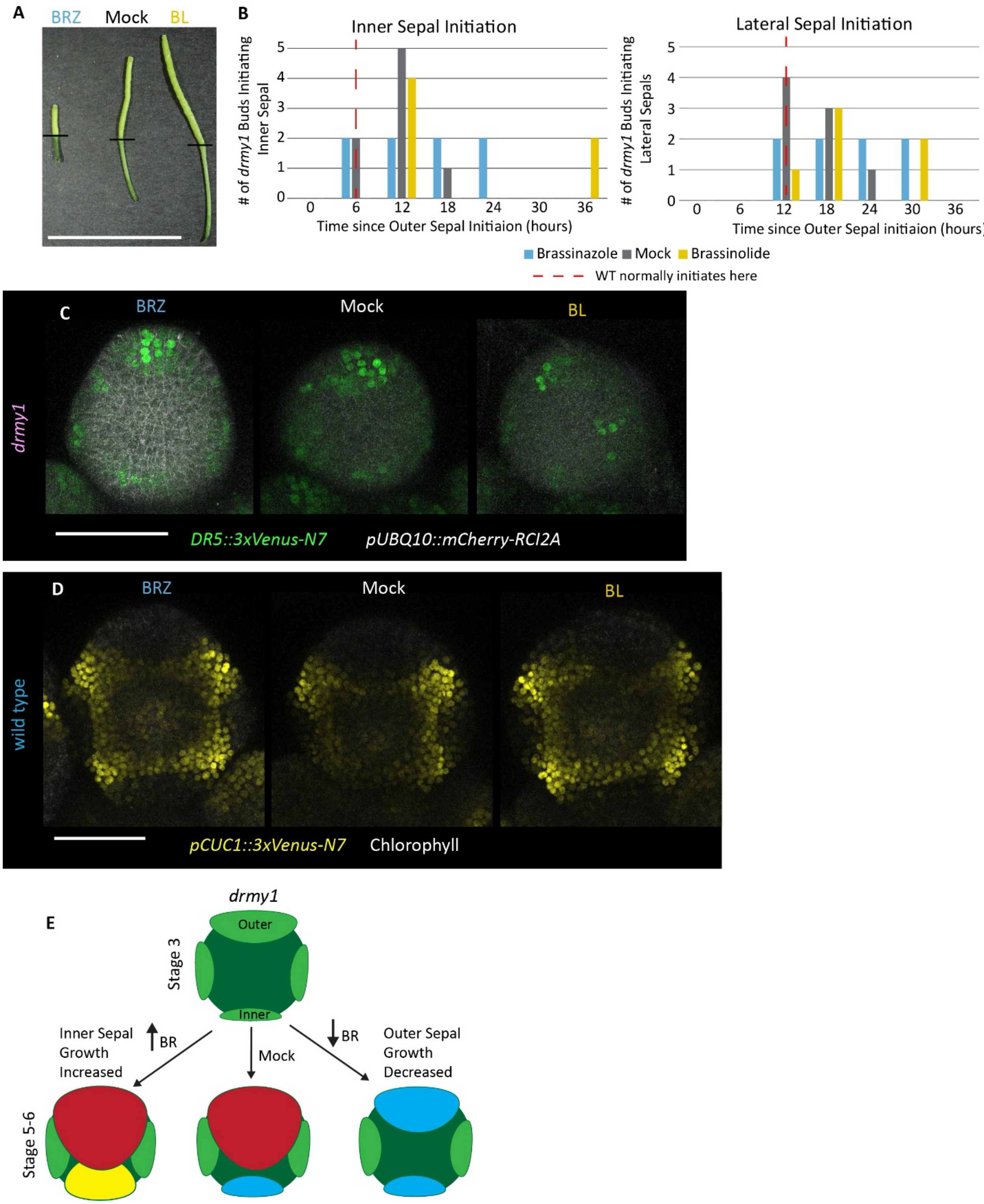

Fig. S7: Altering BR levels does not rescue sepal initiation timing or auxin signaling in *drmy1* and does not alter *CUC1* expression in young flower buds.

A) Early stage 17 siliques just after other floral organs have fallen off the silique 11 days after treatment with 50 μM brassinazole (BRZ), 400 nM brassinolide (BL), or mock. Black lines indicate the pedicel/silique junction. Note both pedicels and silique lengths elongated by BL treatment and shortened by BRZ treatment, as expected.

B) Timing of initiation of the inner and lateral sepals relative to the initiation of the outer sepal in BRZ, BL, and mock treated *drmy1* buds. In WT, inner sepals would normally initiate 6 hours after the outer sepal, and laterals 12 hours after outer sepal (denoted by red dashed lines).

C) *DR5::3xVenus-N7* expression in stage 2 *drmy1* flower buds under BRZ, BL, and mock treatments.

D) *pCUC1::3xVenus-N7* expression in stage 3 WT flower buds under BRZ, BL, and mock treatments.

E) Schematic diagram describing the effect of increasing or decreasing BR signaling on the growth of the inner and outer sepals of *drmy1*.

Scale bars 1 cm (A) or 50 μm (C) and (D).

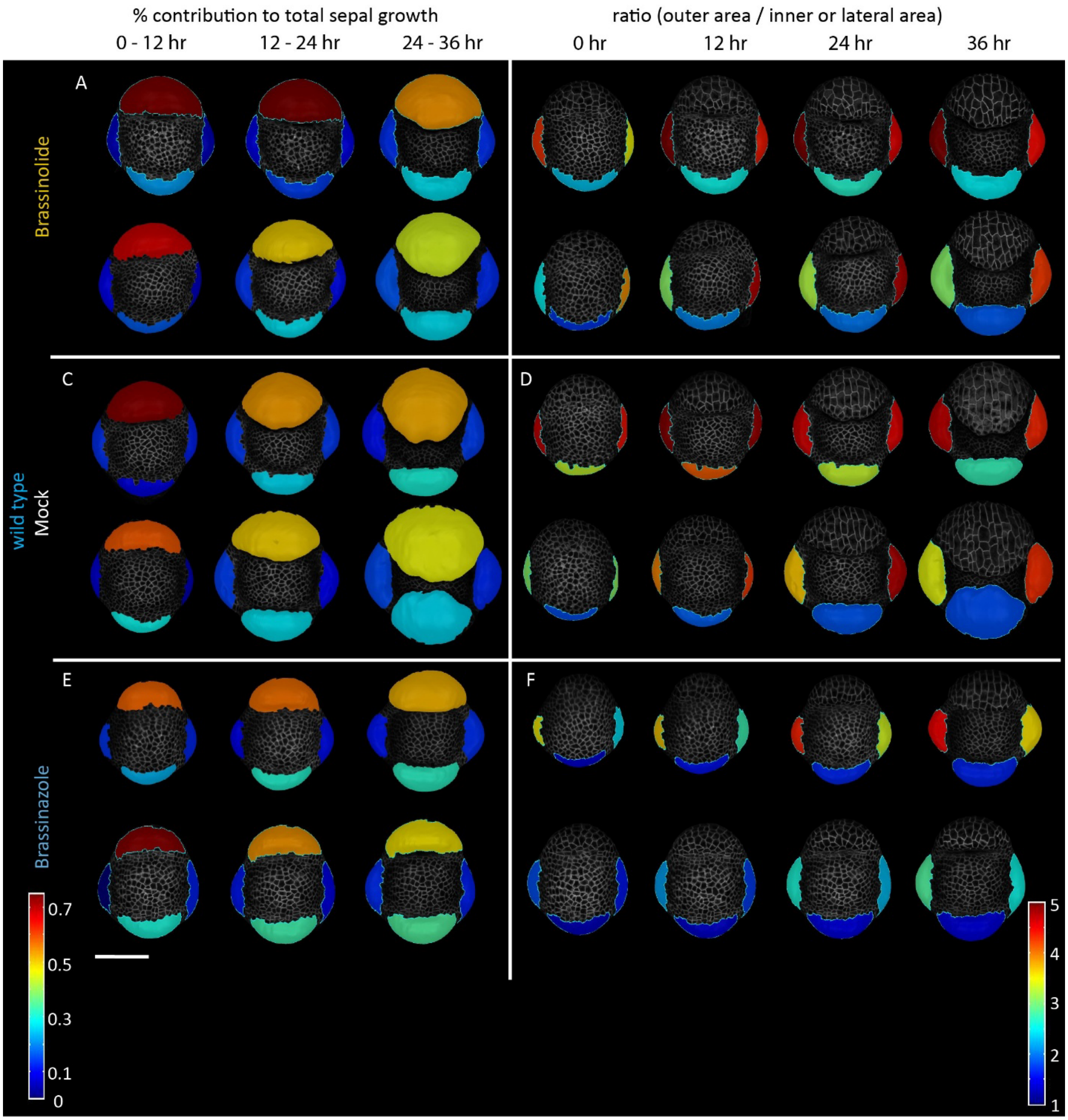

Fig. S8: Live imaging replicates of wild type flower buds.

A, C and E) Proportion of total sepal growth contributed by each sepal in WT bud replicates treated with 400 nM brassinolide (BL) (A), mock (C), or 50 μM brassinazole (BRZ) (E) at 12 hour time intervals.

B, D, and F) Corresponding ratio between the area of the outer sepal to the area of the inner and each lateral sepal under BL (B), mock (D), and BRZ (F) treatments in WT replicates.

Scale bars 50 µm.

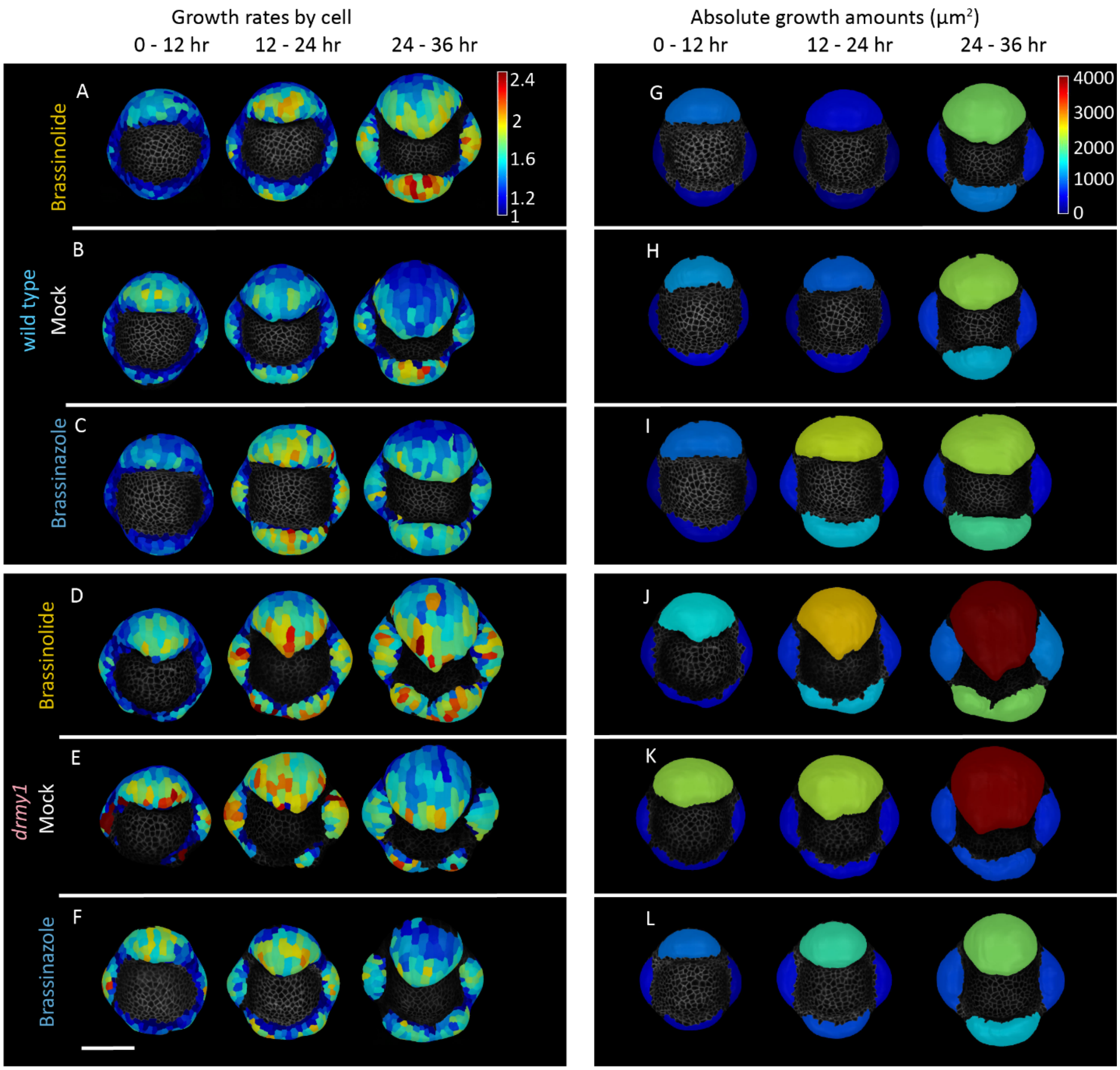

Fig. S9: Absolute growth of sepals and growth rates of individual cells confirms that *drmy1* outer sepal overgrowth is diminished by brassinazole, inner sepal growth increased by brassinolide.

A-C) Growth rates of each cell during each 12 hour interval in WT buds treated with BL (A), mock (B), or BRZ (C). Growth rates are calculated by dividing the area of the cell at the later timepoint by the area in the previous timepoint. Note: these samples do not all correspond to the same samples in G-I.

D-F) Growth rates of each cell during each 12 hour interval in *drmy1* buds treated with BL (D), mock (E), or BRZ (F). Growth rates are calculated by dividing the area of the cell at the later timepoint by the area in the previous timepoint. Note: these samples do not all correspond to the same samples in J-L.

G-I) Absolute growth amounts (in μm^2) during each 12 hour interval in WT buds treated with 400 nM brassinolide (BL) (G), mock (H), or 50 µM brassinazole (BRZ) (I).

J-L) Absolute growth amounts (in μm^2) during each 12 hour interval in *drmy1* buds treated with BL (J), mock (K), or BRZ (L).

Scale bars 50 µm

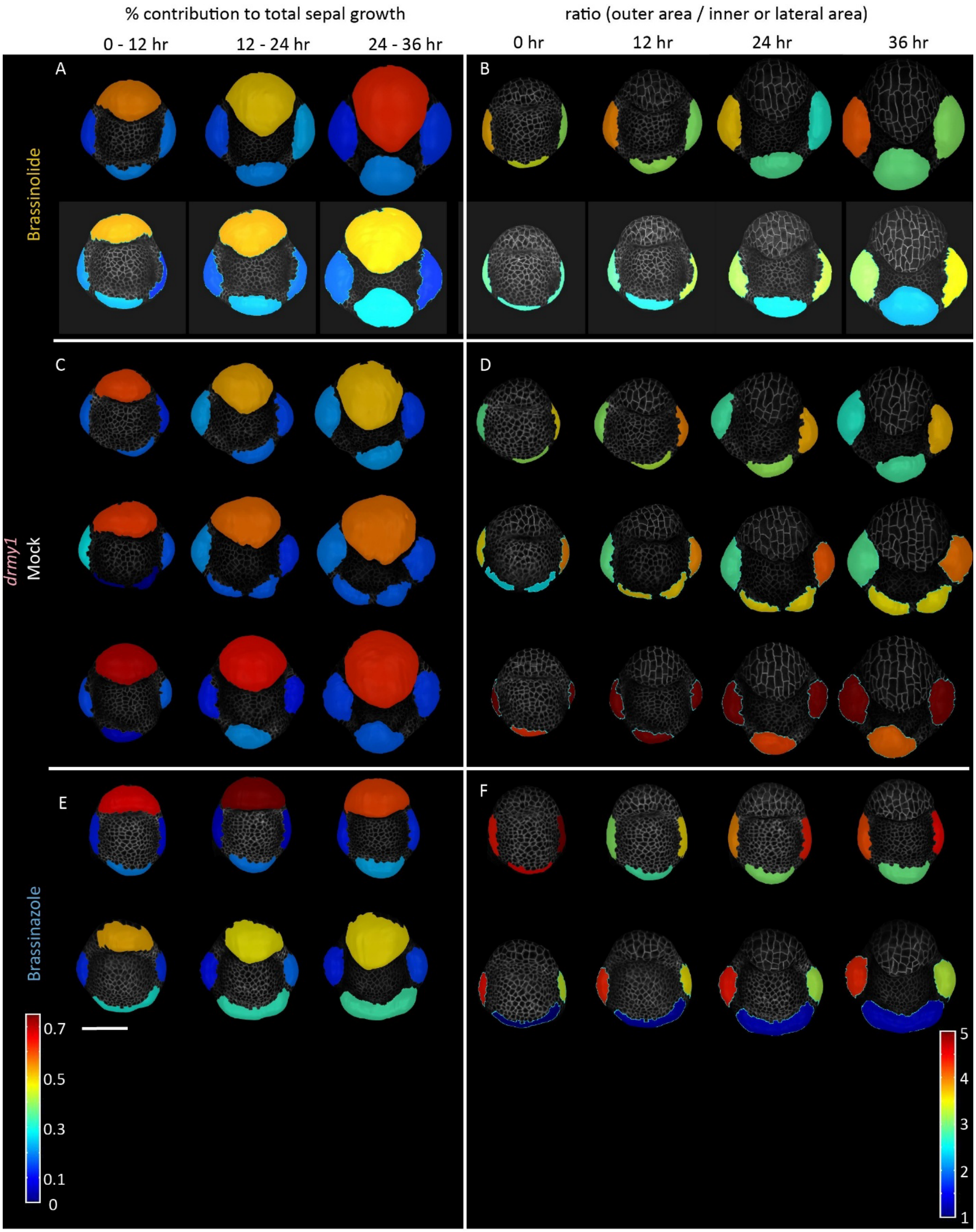

Fig. S10: Live imaging replicates of *drmy1* flower buds.

A, C and E) Proportion of total sepal growth contributed by each sepal in *drmy1* bud replicates treated with 400 nM brassinolide (BL) (A), mock (C), or 50 µM brassinazole (BRZ) (E) at 12 hour time intervals.

B, D, and F) Corresponding ratio between the area of the outer sepal to the area of the inner and each lateral sepal under BL (B), mock (D), and BRZ (F) treatments in *drmy1* replicates. Note the correlation between the proportion of sepal growth coming from the outer sepal and the magnitude of the corresponding outer/inner sepal ratio.

Scale bars 50 µm.

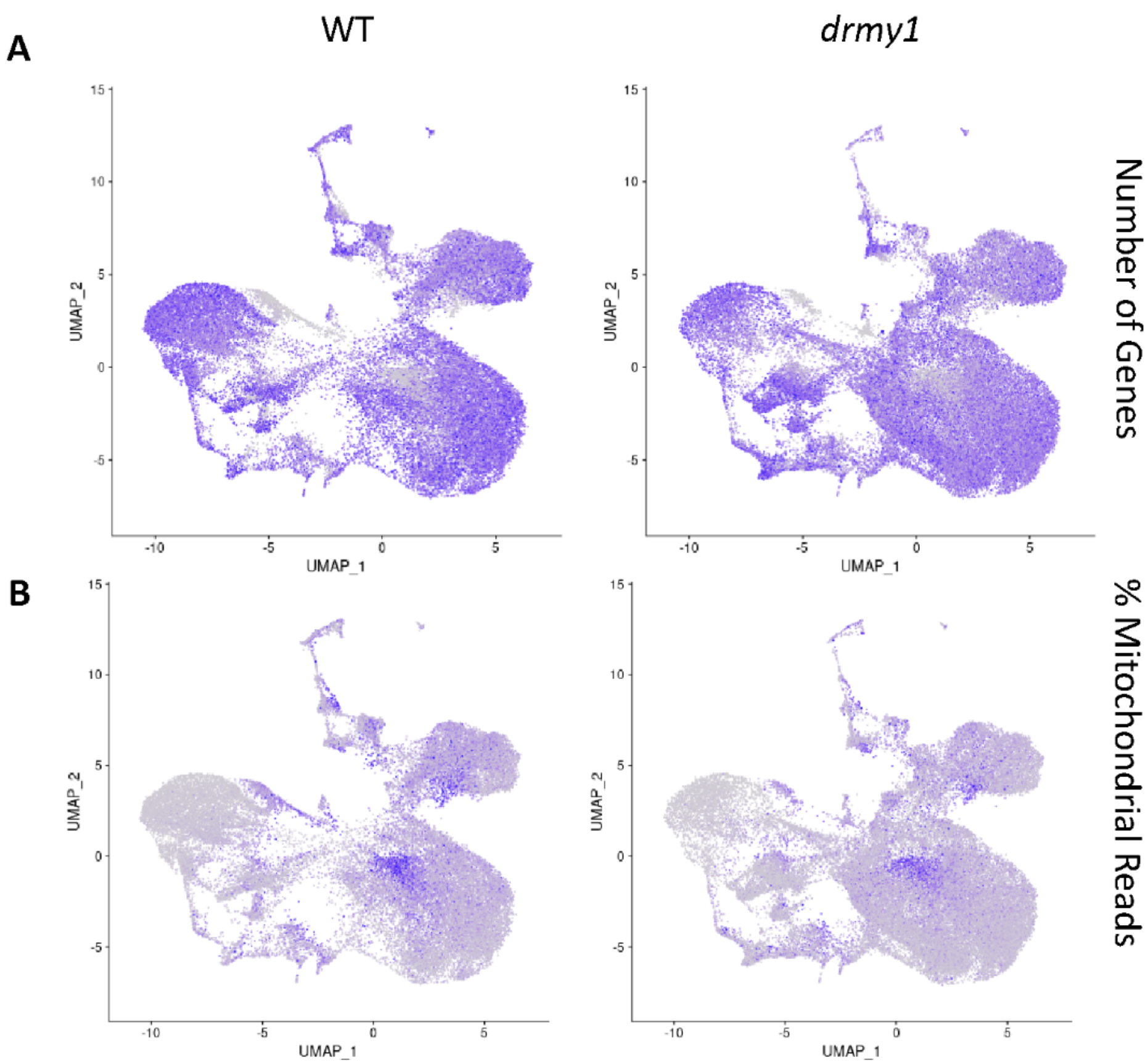

Fig. S11: Mitochondrial and chloroplast genes in single cell RNA-seq.

A-B) Feature plots of various quality control metrics including the total number of genes detected (A) and the percentage of transcripts from mitochondrial genes (B).

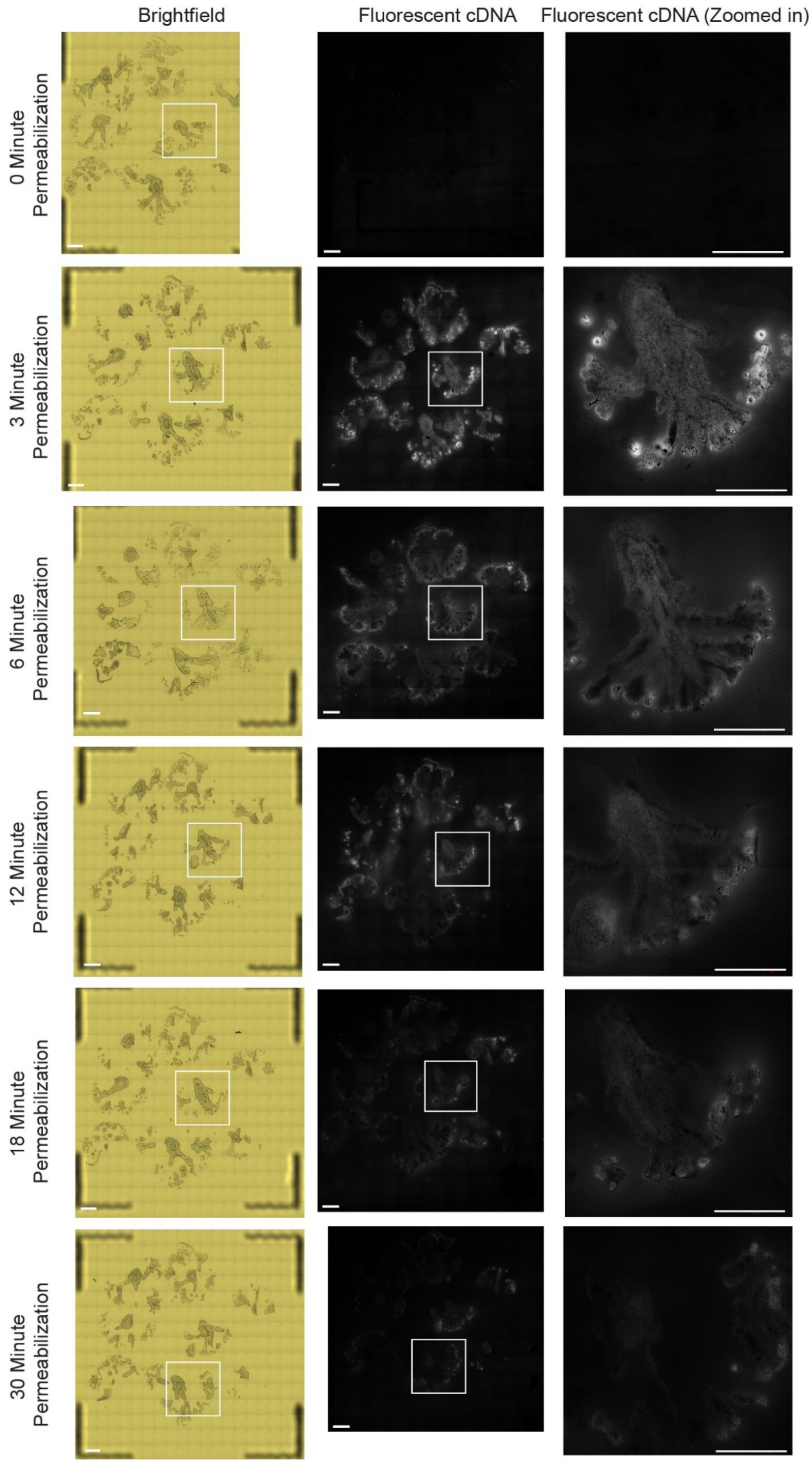
Brightfield
Fluorescent cDNA
Fluorescent cDNA (Zoomed in)
0 Minute Permeabilization
3 Minute Permeabilization
6 Minute Permeabilization
12 Minute Permeabilization
18 Minute Permeabilization
30 Minute Permeabilization

Fig. S12: Optimization of permeabilization times indicates 3-5 minutes yields the best RNA signal. Stitched brightfield and confocal images of the capture areas and of selected areas. More fluorescent cDNA signal represents better RNA release from permeabilization.

Scale bars 500 µm

Additional file 2: Table S1: List of genes with >3% variance explained by cell cycle phase and removed from single cell RNAseq dataset
Table S2: List of markers used to help identify single cell RNAseq clusters
Gene_symbol: Short name of gene:
Locus: Name of each gene locus
Location: A brief description of the cell type, function, or signaling pathway that the gene is involved in
Reference: What publication, if any, we pulled information from for each gene. Zhang = Zhang et al. (2021), Conde = Conde et al. (2022), neumann = Neumann et al. (2022), noref = no reference
Notes: brief notes about some of the markers

Additional file 3: Table S3: Output of DEseq2 differential gene expression analysis on pseudobulked scRNA-seq dataset.
Includes Locus (gene identifier), Short name (gene name), and relevant statistics (including log 2-fold change, p-value, and adjusted p-value) for each gene. Each tab represents a single cell cluster (e.g “0” tab refers to cluster 0, etc.)

Additional file 4: Table S4: Output of Gene Set Enrichment Analysis (GSEA) for gene ontology (GO) term enrichment.
Includes GO term, set size, enrichment score, and relevant statistics (including p-value, adjusted p-values, q-value, rank, etc.). Each tab represents a single cell cluster (e.g “0” tab refers to cluster 0, etc.).

Additional file 5: Table S5: List of genes from Nemhauser et al. (2006) up and downregulated by application of each hormone.
Lists have been filtered to include only genes present in our single cell dataset.
Table S6: Statistical information for dotplots in Figure S3A, Figures 3B, C, S3B, Figure 4H, and Figure S3C.
Seurat_clusters: identifies which seurat cluster each row of data pertains to.
pval: the p-value determined by Wilcoxon's ranked sums test comparing SCT normalized expression values of the gene indicated in the "gene" column in WT cells vs *drmy1* cells
Log(1/pval): log of the inverse of the p-value. Done for visualization purposes, so that lower p-values appear as bigger dots on the dotplot.
Mean_diff: the difference in the mean expression of each gene in WT minus the expression in *drmy1*.
mean_pct_change: Mean_diff divided by the mean of WT.
mean_pct_change_visualize: the same as mean_pct_change, except any samples where the p-value exceeded the Bonferroni adjusted p-value thresholds of $p < 0.00014$ had

their mean_pct_change_visualizeset to zero. This was done for visualization purposes, so that in the dotplot figure any comparisons that did not reach significance thresholds would be colored white and thus not be visible against the background, so the reader does not draw any conclusions from these insignificant samples.
gene: name of gene being compared
Table S7: Output of Seurat FindConservedMarkers to identify markers for cluster 2 (boundary cells). Includes gene name and relevant statistics – max p-value used to rank the top 100 markers for module score calculation.
Table S8: ANOVA tables and Tukey's Honest Significant Difference (HSD) test results from figure 5.
Table S9: A list of cell cycle marker genes used to annotate the cell cycle phase of each cell, based on data from Zhang et al. (2021)
Locus: Gene locus
Phase: What phase of the cell cycle this gene is a marker for
Symbol: short name for the gene, where applicable.
Araport11: A short description of the gene based off of annotations from Araport11
Function: A brief description of the function of each gene, where known.
Table S10: Output of ScoreMarkers used to identify additional single cell cluster specific marker genes. Includes gene name, single cell cluster that gene is a marker for, and relevant statistics. A minimum threshold of 0.7 for mean.AUC was used to filter markers.

Additional File 6: Table S11: Metadata from single cell-RNAseq (Seurat format).
